# The role of the *msaABCR* operon in implant-associated chronic osteomyelitis in *Staphylococcus aureus* USA300 LAC

**DOI:** 10.1186/s12866-020-01964-8

**Published:** 2020-10-27

**Authors:** Gyan S. Sahukhal, Michelle Tucci, Hamed Benghuzzi, Gerri Wilson, Mohamed O. Elasri

**Affiliations:** 1 Present Address: Center for Molecular and Cellular Biosciences, The University of Southern Mississippi, 118 College Drive # 5018, Hattiesburg, MS 39406 USA; 2 Department of Orthopaedics, University of Mississippi Medical Center, Jackson, MS USA

**Keywords:** *Staphylococcus aureus*, *msaABCR* operon, Osteomyelitis, K-wire implants, Virulence factors

## Abstract

**Background:**

The *msaABCR* operon regulates several staphylococcal phenotypes such as biofilm formation, capsule production, protease production, pigmentation, antibiotic resistance, and persister cells formation. The *msaABCR* operon is required for maintaining the cell wall integrity via affecting peptidoglycan cross-linking. The *msaABCR* operon also plays a role in oxidative stress defense mechanism, which is required to facilitate persistent and recurrent staphylococcal infections. *Staphylococcus aureus* is the most frequent cause of chronic implant-associated osteomyelitis (OM). The CA-MRSA USA300 strains are predominant in the United States and cause severe infections, including bone and joint infections.

**Results:**

The USA300 LAC strain caused significant bone damage, as evidenced by the presence of severe bone necrosis with multiple foci of sequestra and large numbers of multinucleated osteoclasts. Intraosseous survival and biofilm formation on the K-wires by USA300 LAC strains was pronounced. However, the *msaABCR* deletion mutant was attenuated. We observed minimal bone necrosis, with no evidence of intramedullary abscess and/or fibrosis, along reduced intraosseous bacterial population and significantly less biofilm formation on the K-wires by the *msaABCR* mutant. microCT analysis of infected bone showed significant bone loss and damage in the USA300 LAC and complemented strain, whereas the *msaABCR* mutant’s effect was reduced. In addition, we observed increased osteoblasts response and new bone formation around the K-wires in the bone infected by the *msaABCR* mutant. Whole-cell proteomics analysis of *msaABCR* mutant cells showed significant downregulation of proteins, cell adhesion factors, and virulence factors that interact with osteoblasts and are associated with chronic OM caused by *S. aureus*.

**Conclusion:**

This study showed that deletion of *msaABCR* operon in USA300 LAC strain lead to defective biofilm in K-wire implants, decreased intraosseous survival, and reduced cortical bone destruction. Thus, *msaABCR* plays a role in implant-associated chronic osteomyelitis by regulating extracellular proteases, cell adhesions factors and virulence factors. However additional studies are required to further define the contribution of *msaABCR*-regulated molecules in osteomyelitis pathogenesis.

## Background

*Staphylococcus aureus* is the most common pathogen causing chronic infections in humans [1–3], and osteomyelitis (OM) is one of the primary chronic infections caused by this pathogen [4–7]. According to Gerber et al. [2] and Weichert et al. [8], approximately 2.5 of every 1000 hospital admitted children has staphylococcal OM [2, 8]. Among adults, staphylococcal OM frequently complicates open fractures and soft-tissue infections [6, 9]. In addition, nosocomial infections by *S. aureus* in immunocompromised patients, severely debilitated patients, and patients with indwelling medical devices are on the rise [10–12]. The increasing use of surgically implanted orthopedic medical devices has led to an increased risk of implant-associated OM infection [6, 13, 14]. Implant-associated OM is a chronic and deep bone infection, and studies have shown that *S. aureus* is the most common causative pathogen, accounting for 20–30% of chronic OM cases associated with fracture fixation and prosthetic joint replacement [15–18]. According to Darouiche [19], 600, 000 artificial joint replacements and 2 million fracture fixation devices are implanted each year in the U.S., resulting in > 110,000 infections [19]. Furthermore, 65% of military injuries are orthopedic, with an infection rate of 50%, and *S. aureus* is the most prevalent pathogen in these infections [19]. The community-associated, methicillin-resistant *S. aureus* (CA-MRSA) strains are emerging as leading pathogens causing the severe sepsis associated with acute OM [5, 7, 8, 20]. The CA-MRSA USA300 clone is the predominant strain in the United States and has been responsible for severe infections, including bone and joint infections [21–24].

Treatment of OM infections by *S. aureus* is complex and involves one or more surgical debridement, implant removal, and prolonged antimicrobial therapy. The ability of *S. aureus* to form biofilm, small colony variants, and persister cells inside osteoblasts contributes to treatment recalcitrance [25–28]. Previous retrospective studies by Marculescu et al. [29] and Lee et al. [30] both found that the treatment failure rate for implant-associated OM is high (58.2 and 41.8%, respectively). Thus, despite all the sophisticated procedures and efforts to maintain high sterility standards to prevent onset of implant-associated OM infection, it is still a significant challenge in the clinic [11, 12].

The *msaABCR* operon is a four-gene operon that regulates several virulence factors (proteases as well as factors involved in pigmentation, toxin production, and capsule production), biofilm formation, persister cell formation, oxidative stress response, and antibiotic susceptibility in *S. aureus* [31–37]. We also showed that *msaABCR* deletion mutants are not defective in growth [33]. Previously, we showed that *msaABCR* mutants are defective in biofilm formation due to increased production of extracellular proteases in vitro [31]. MsaB is the only protein expressed from this operon, and we have shown that MsaB is the DNA-binding protein that directly regulates the virulence factor for processes such as capsule production [32, 34, 35]. A separate study by Caballero et al. [38] showed that MsaB is an RNA chaperone that positively and negatively modulates the expression of its target proteins, which are involved in carbohydrate metabolism, ribonucleotide metabolism, and the stress response, and these include several other virulence factors [38]. Previously, we also showed that the *msaABCR* operon regulates the expression of 238 genes that are involved in metabolism, pathogenesis, transport, signal transduction, and stress response [33]. The primary objective of this study is to test the contribution of the global modulator *msaABCR* operon in pathogenesis in vivo using implant-associated OM in a SD rat model. In this model of implant associated osteomyelitis, we used K-wires coated with *S. aureus* strains to implant transcortically through the tibial metaphysis as previously described to mimic clinical OM infection [39, 40]. We used community-associated methicillin-resistant *S. aureus* strain USA300 LAC and its isogenic *msaABCR* mutant.

## Results

### The chronic implant-associated OM model

**Fig. 1:**
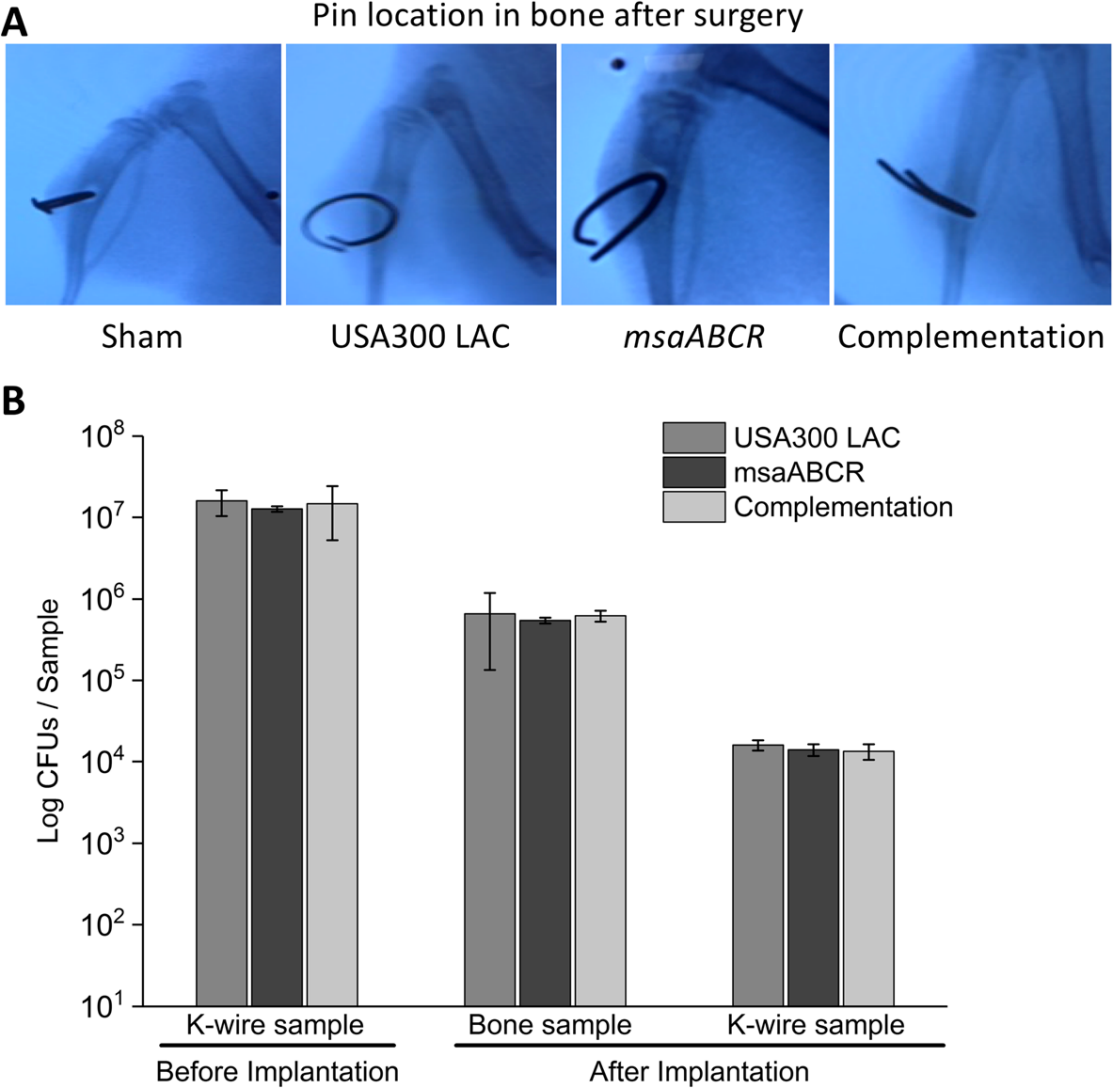
Examination of K-wire localization and microbial load in bone and K-wires after surgery. The K-wire was inserted in the tibia of Sprague Dawley rats surgically and examined by X-ray to assure its positioning (**a**). The microbial load before K-wire implantation and after surgery (**b**). The microbial load was estimated for both K-wires and bone (*n* = 3) to ensure an equal starting microbial load (USA300 LAC, *msaABCR* mutant, and complementation strains) prior to the infection process. Error bars indicate standard errors of the means. The differences in the numbers of microbial CFUs between the test strains were not statistically significant when analyzed by one-way ANOVA followed a post-hoc Tukey test

Sprague Dawley rats were used to study the role of the *msaABCR* operon in the pathogenesis of chronic implant-associated OM and intraosseous bacterial survival. Rats were infected with a clinical isolate of USA300 LAC due to its prevalence in community-associated invasive infections. For induction of chronic implant-associated OM, K-wires were first colonized with the test strains (USA300 LAC, *msaABCR* mutant, and *msaABCR* complementation) and implanted transcortically through the bone metaphysis (Fig. 1a). To ensure that the bacterial cells attach the K-wires at the same rate, the bacterial CFUs were enumerated from the colonized K-wires (six K-wires per bacterial strain). The average number of bacterial CFUs colonized in the K-wires (1.25X10^7^ CFUs) was similar in all test strains and were not statistically different (Fig. 1b). Also, to ensure an equivalent number of bacterial inoculums in the bone prior to infection process and during surgery, the infected K-wires with test strains (3 animals per group) were inserted in the rat bone. The rats were humanely euthanized immediately to harvest the bone and K-wire samples to enumerate the bacterial CFUs. The average bacterial load retained in the bone (6.09X10^5^ CFUs) and on the K-wires (1.45X10^4^ CFUs) after the surgical procedure were not statistically different from each other (Fig. 1b).

### Intraosseous bacterial burden and biofilm formation

**Fig. 2:**
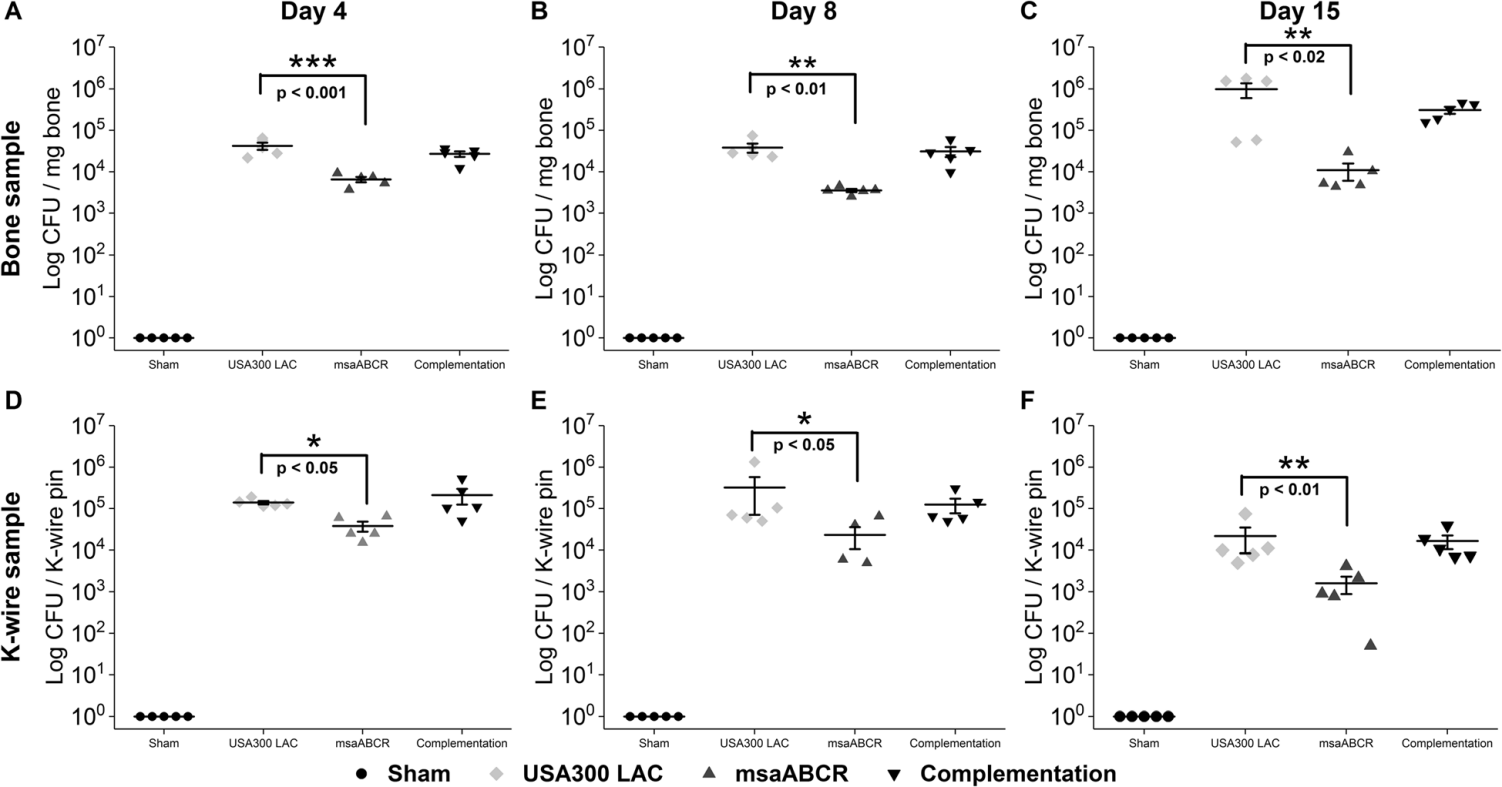
Microbial count from bone and K-wires samples. Microbial enumeration from post-mortem bone samples (**a-c**) and K-wire samples (*n* = 5) were obtained to determine intraosseous bacterial survivability and biofilm formation on the K-wires (**d-f**) on days 4, 8, and 15 post-infection (left to right). The infected bone sample and K-wires were harvested after each infection period (days 4, 8, and 15). Error bars indicate standard errors of the means. The bacterial CFUs difference between the USA300 LAC and *msaABCR mutant* strains was analyzed using one-way ANOVA followed by a post-hoc Tukey test. A *P*-value of < 0.05 was considered statistically significant (*p-value < 0.05, **p-value < 0.01, and ***p-value < 0.001)

The intraosseous bacterial burden in the infected bone was assessed after each infection period as described in the methods (Fig. 2a, b, and c). The K-wires were carefully excised from the infected bone samples and processed for microbial counting to quantify biofilm formation in the K-wires (Fig. 2d, e, and f). The results showed that the intraosseous bacterial burden in the infected bone and biofilm formation in the k-wires were significantly less in the *msaABCR* mutant group compared to the USA300 LAC and the complementation group during all post-infection periods (Fig. 2). These findings showed that the *msaABCR* mutant is defective in intraosseous survival and biofilm formation on K-wires in the implant-associated OM rat model (Fig. 2). These observations correlate with our previous findings showing a defective biofilm phenotype for the *msaABCR* mutant under in vitro conditions [31].

### Dissemination of *Staphylococcus* to different organs

**Fig. 3:**
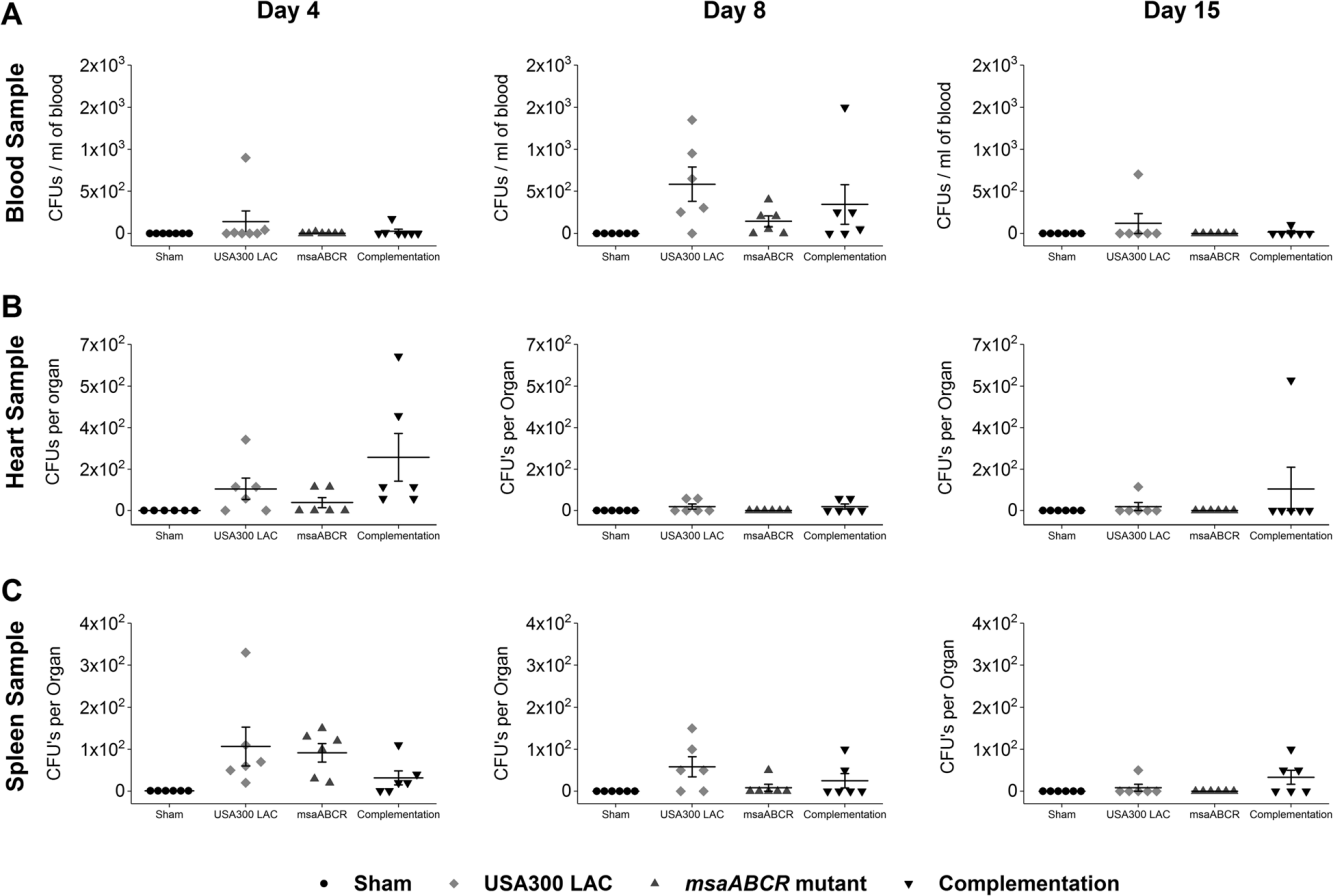
Microbial count from blood, heart, and spleen samples (left to right). The infected blood (**a**), heart (**b**), and spleen (**c**) samples were processed to enumerate the bacterial CFUs, as described in methods, after each infection period (on days 4, 8, and 15). Error bars indicate standard errors of the means. The difference in microbial counts between the USA300 LAC and *msaABCR* mutant strains was analyzed using one-way ANOVA followed by a post-hoc Tukey test. A *P*-value of < 0.05 was considered statistically significant

To study bacterial survival in blood and dissemination to different vital organs (heart, liver, spleen, lungs, kidney, and liver), microbial counts were obtained from the post-mortem blood and organ samples. Our results showed that the bacterial CFUs in blood, heart, and spleen samples recovered from the animals infected with the *msaABCR* mutant were less compared to USA300 LAC and complementation strains during all post-infection periods, but the differences were not statistically significant (Fig. 3a, b, and c). However, in the liver, kidney, and lung samples, all the animals infected with all three test strains showed similar numbers of bacterial CFUs throughout the infection periods (Additional file 1: Fig. S1).

### Analysis of bone damage by microCT

To further delineate the role of the *msaABCR* operon in the pathogenesis of OM, the infected bones harvested after each infection period were analyzed by microCT.

**Fig. 4:**
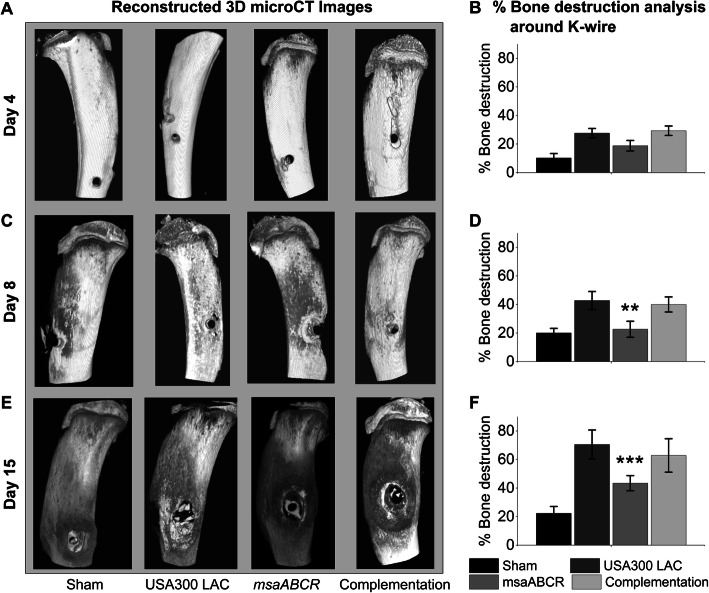
Reconstructed 3D microCT images of bone samples (sham and infected) and cortical bone destruction analysis. The sham and infected bone samples were harvested after each infection period (on days 4, 8, and 15). The 3D microCT images of sham and infected bone samples (**a, c**, and **e**) and cortical bone destruction analysis of sham and infected bones samples **(b, d,** and **f**). Reconstructed 3D images showed significant bone damage and/or loss by USA300 LAC and complementation strains, which was also revealed by cortical bone destruction analysis. Bone destruction analysis was subjected to one-way ANOVA followed by a post-hoc Tukey test. Error bars indicate standard errors of the means. A *P*-value of < 0.05 was considered statistically significant (**p-value < 0.01, and ***p-value < 0.001)

**Fig. 5:**
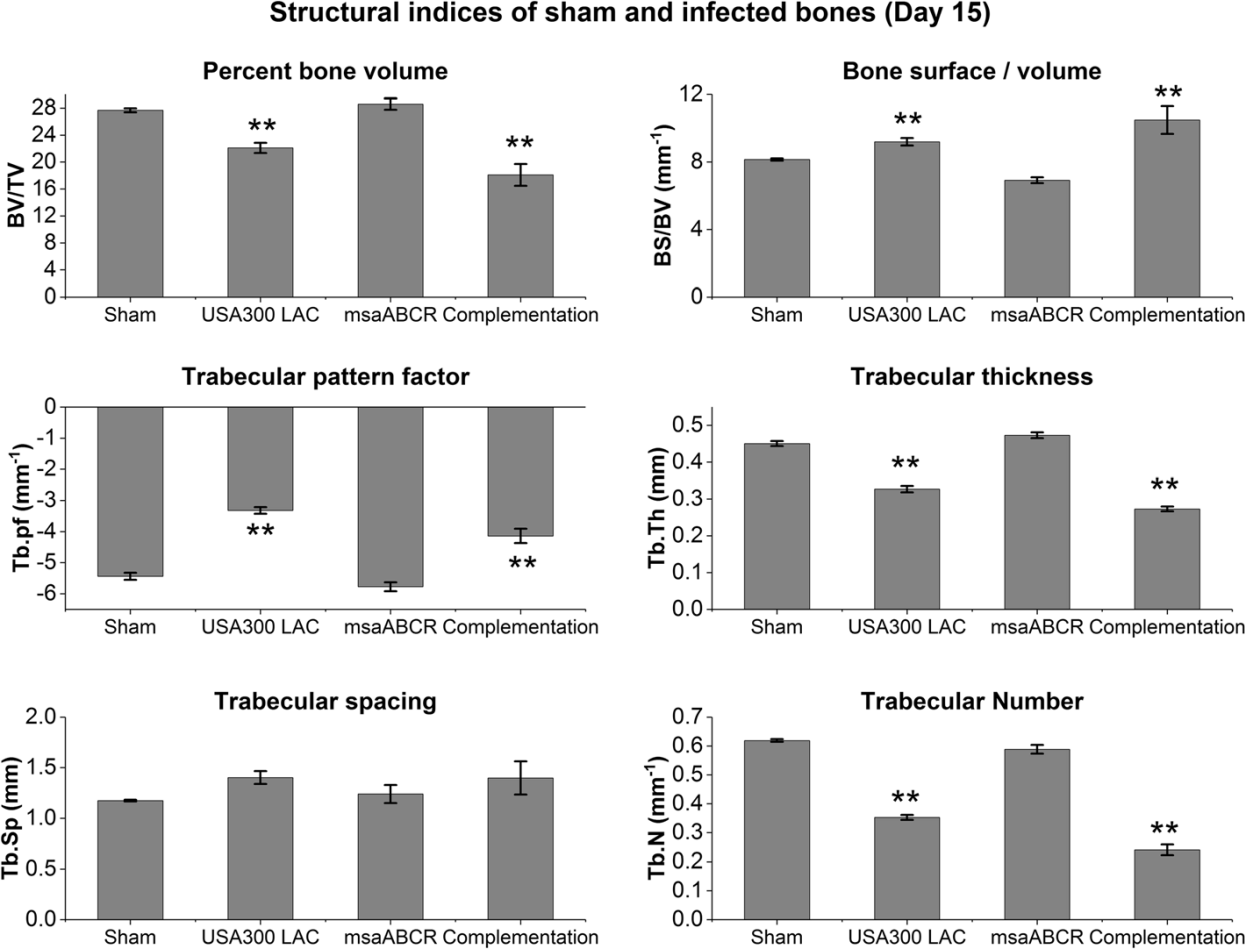
Structural indices of sham and infected bones after day 15 post-infection. Analysis of trabecular bone microarchitecture (percentage bone volume, BV/TV; bone surface/volume ratio, BS/BV; trabecular pattern factor, Tb.Pf; trabecular thickness, Tb.Th; trabecular spacing, Tb.Sp; and trabecular number, Tb.N) were performed. All values were analyzed using one-way ANOVA followed by a post-hoc Tukey test. Error bars indicate standard errors of the means. A *P*-value of < 0.05 was considered statistically significant (**p-value < 0.01)

At day 4 post-infection, no significant bone damage was observed in the reconstructed 3D microCT images of infected bone by all the test strains (Fig. 4a and b). However, we observed significant cortical bone damage around the K-wire insertion area in bone infected with the USA300 LAC and complementation strains after 8- and 15- days post-infection periods relative to the bone infected with the *msaABCR* mutant strains (Fig. 4c-f). During the process of harvesting K-wires before microCT scanning of the *msaABCR* mutant infected bone (Day 8), a small piece of bone was missing near the infection area, as evident in the 3D image (Fig. 4c). Morphometric analysis showed no significant changes in bone microarchitectural patterns in the infected bones by all test strains after 4- and 8- days post-infection (Additional file 2: Fig. S2 and Additional file 3: Fig. S3). However, the significant changes the bone microarchitectural patterns in the bone infected with the USA300 LAC and complementation strains were evident after 15 days post-infection. We observed significantly altered BV/TV, BS/BV, Tb.pf, Tb. Sp, Tb. Th, and Tb. N in the bone samples infected with the USA300 LAC and complementation strains, thus revealing compromised trabecular microarchitecture and massive bone destruction by these strains relative to the *msaABCR* mutant strains (Fig. 5).

### Histopathological analysis of infected bone

The infected bone samples were fixed, sectioned, and stained with H&E, and histopathological scores were assessed [41] , as shown in Table 1.

**Table 1:** Histopathological parameters and scoring system to evaluate bone damage as previously described by Smeltzer et al. [41]

Histopathological parameters and scoring	
**Intraosseous acute inflammation (IAI):**	
0 Not present	
1 Minimal-to-mild inflammation with no intramedullary abscess	
2 Moderate-to-severe inflammation with no intramedullary abscess	
3 Minimal-to-mild inflammation with intramedullary abscess	
4 Moderate-to-severe inflammation with intramedullary abscess	
**Intraosseous chronic inflammation (ICI):**	
0 Not present	
1 Minimal-to-mild chronic inflammation with no significant intramedullary fibrosis	
2 Moderate-to-severe chronic inflammation with no significant intramedullary fibrosis	
3 Minimal-to-mild chronic inflammation with significant intramedullary fibrosis	
4 Moderate-to-severe chronic inflammation with significant intramedullary fibrosis	
**Periosteal inflammation (PI):**	
0 Not present	
1 Minimal-to-mild inflammation with no subperiosteal abscess formation	
2 Moderate-to-severe inflammation with no subperiosteal abscess formation	
3 Minimal-to-mild inflammation with subperiosteal abscess formation	
4 Moderate-to-severe inflammation with subperiosteal abscess formation	
**Bone necrosis (BN):**	
0 No evidence of necrosis	
1 Single focus of necrosis without sequestrum formation	
2 Multiple foci of necrosis without sequestrum formation	
3 Single focus of sequestrum	
4 Multiple foci of sequestra	

#### Day 4 post-infection

**Fig. 6:**
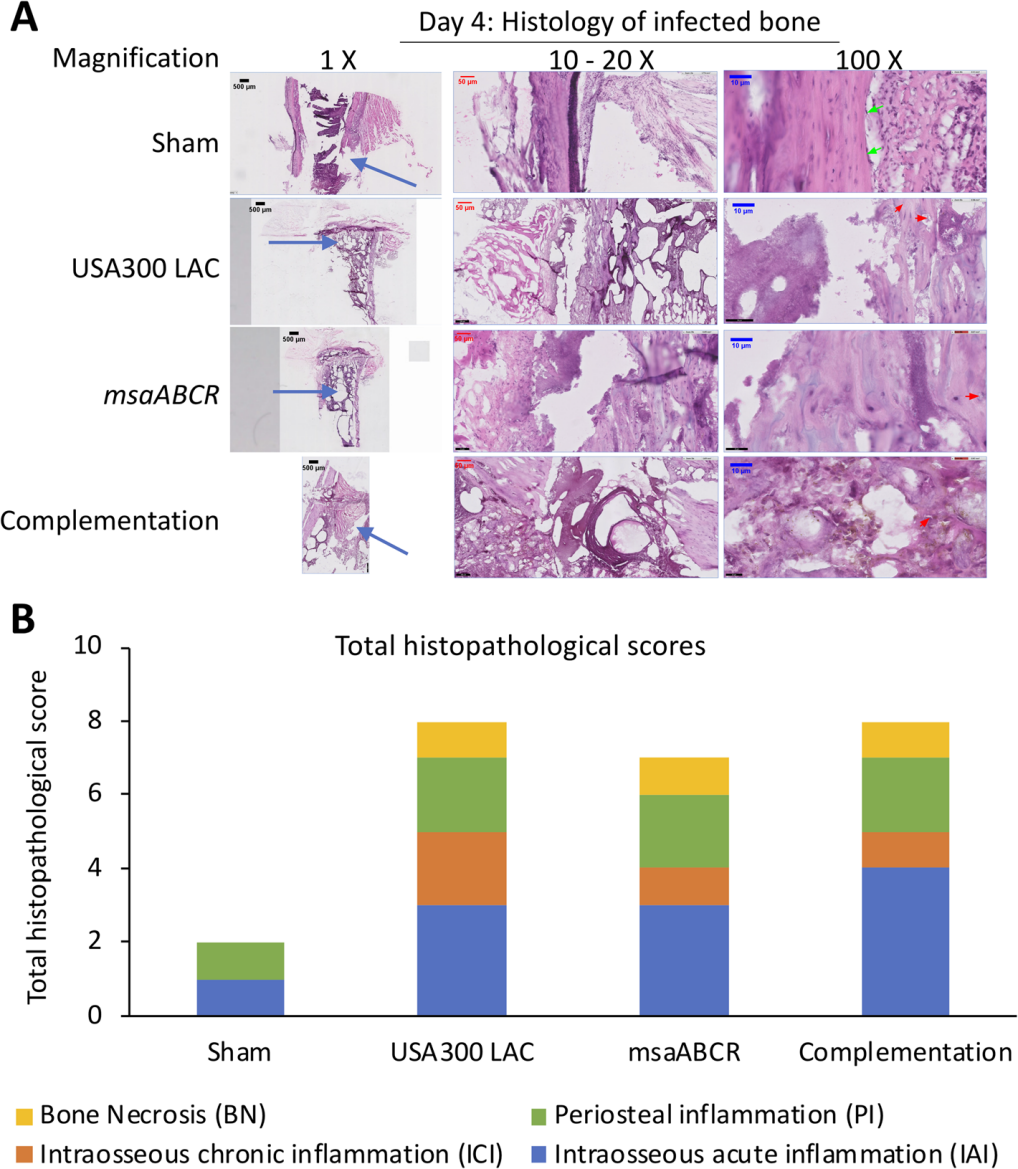
Hematoxylin and eosin (H&E) stained sections of representative bone samples after day 4 post-infection. Bone infected with sham (control) pins or with the USA300 LAC (wild type), *msaABCR mutant*, or complementation strains (**a**) at low (left) and high (right) magnification. Blue arrows show the points of insertion of K-wires, green arrows show osteoblast cells, and red arrows show osteoclasts cells. Total histopathological score of infected bone (**b**). Histopathological scores were assessed on the basis of intraosseous acute inflammation (IAI, blue), intraosseous chronic inflammation (ICI, orange), periosteal inflammation (PI, green), and bone necrosis (BN, yellow)

Bone samples from the sham group showed a minimal presence of intraosseous inflammatory cells and intraosseous fibrosis, with no evidence of any periosteal abscess or bone necrosis (Fig. 6a). Bone infected with the USA300 LAC strain showed severe intraosseous inflammation and evidence of intramedullary abscess. We also observed the presence of osteoclast activity in bone infected with USA300 LAC strain (Fig. 6a). Bone infected by the *msaABCR* mutant showed a minimal-to-mild intraosseous inflammatory response with intramedullary abscess (Fig. 6a). Bone infected with complementation strain showed severe intraosseous inflammation and evidence of intramedullary abscess (Fig. 6a). Overall, bone harvested from the sham group received a total histopathological score of 2, whereas bone infected by USA300 LAC, *msaABCR* mutant, and complementation strains received total histopathological scores of 8, 7, and 8, respectively (Fig. 6b).

#### Day 8 post-infection

**Fig. 7:**
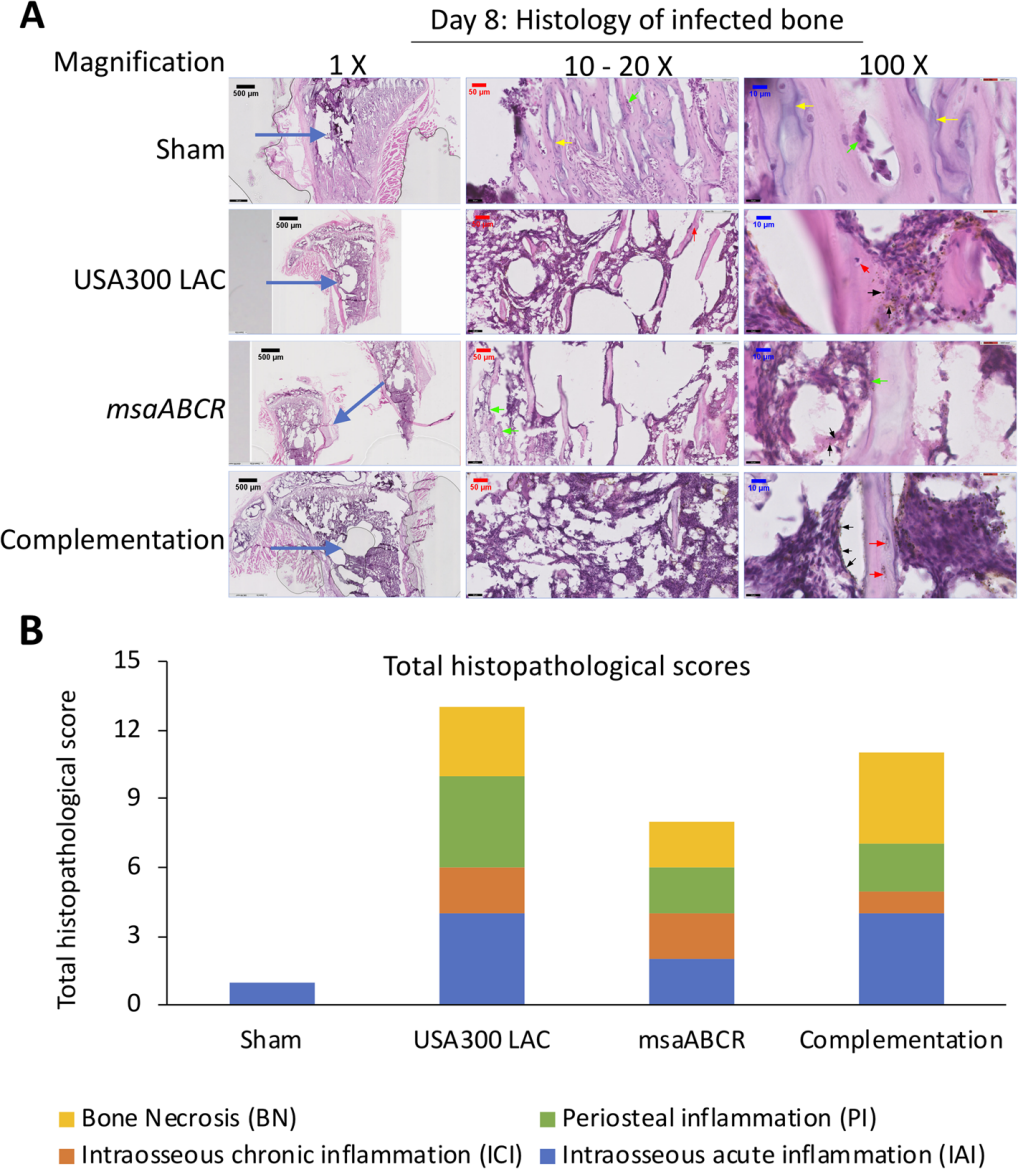
Hematoxylin and eosin (H&E) stained sections of representative bone samples after day 8 post-infection. Bone infected with sham (control) pins or with the USA300 LAC, *msaABCR mutant*, or complementation strains (**a**) at low (left) and high (right) magnification. Blue arrows show the points of insertion of K-wires, green arrows show osteoblast cells, red arrows show osteoclasts cells, black arrows show bacterial cells, and yellow arrows show newly formed bones. Total histopathological score of infected bone (**b**). Histopathological scores were assessed on the basis of intraosseous acute inflammation (IAI, blue), intraosseous chronic inflammation (ICI, orange), periosteal inflammation (PI, green), and bone necrosis (BN, yellow)

We observed increased osteoblast response, with a minimal presence of intraosseous inflammatory cells, and with no evidence of intramedullary abscess, intraosseous fibrosis, periosteal abscess, and bone necrosis in the bone infected with sham group (Fig. 7a). The presence of moderate-to-severe intraosseous and periosteum inflammation with evidence of intramedullary abscess as well as bone necrosis with multiple foci of sequestra are the hallmark features of bone infected with the USA300 LAC and complementation strains (Fig. 7a). An increased osteoblast response lining the site adjacent to the K-wire was also observed in the bone infected with the *msaABCR* mutant, but not observed in the bones infected with USA300 LAC and complementation strains (Fig. 7a). However, we did observe moderate-to-severe intraosseous inflammation, minimal-to-mild intraosseous fibrosis and a moderate-to-severe inflammation without any evidence of intramedullary abscess and periosteal abscess in the bone infected with *msaABCR* mutant (Fig. 7a). Bone necrosis with multiple foci without any sequestrum was observed in bone infected with the *msaABCR* mutant (Fig. 7a). In addition, we also observed traces of bacteria in the bones infected by each of the test strains, which was not evident in the bones infected on days 4 and 15. Overall, on day 8 after infection, the sham bone and bones infected with USA300 LAC, *msaABCR* mutant, and complementation strains received histopathological scores of 1, 13, 8, and 11, respectively (Fig. 7b).

#### Day 15 post-infection

**Fig. 8:**
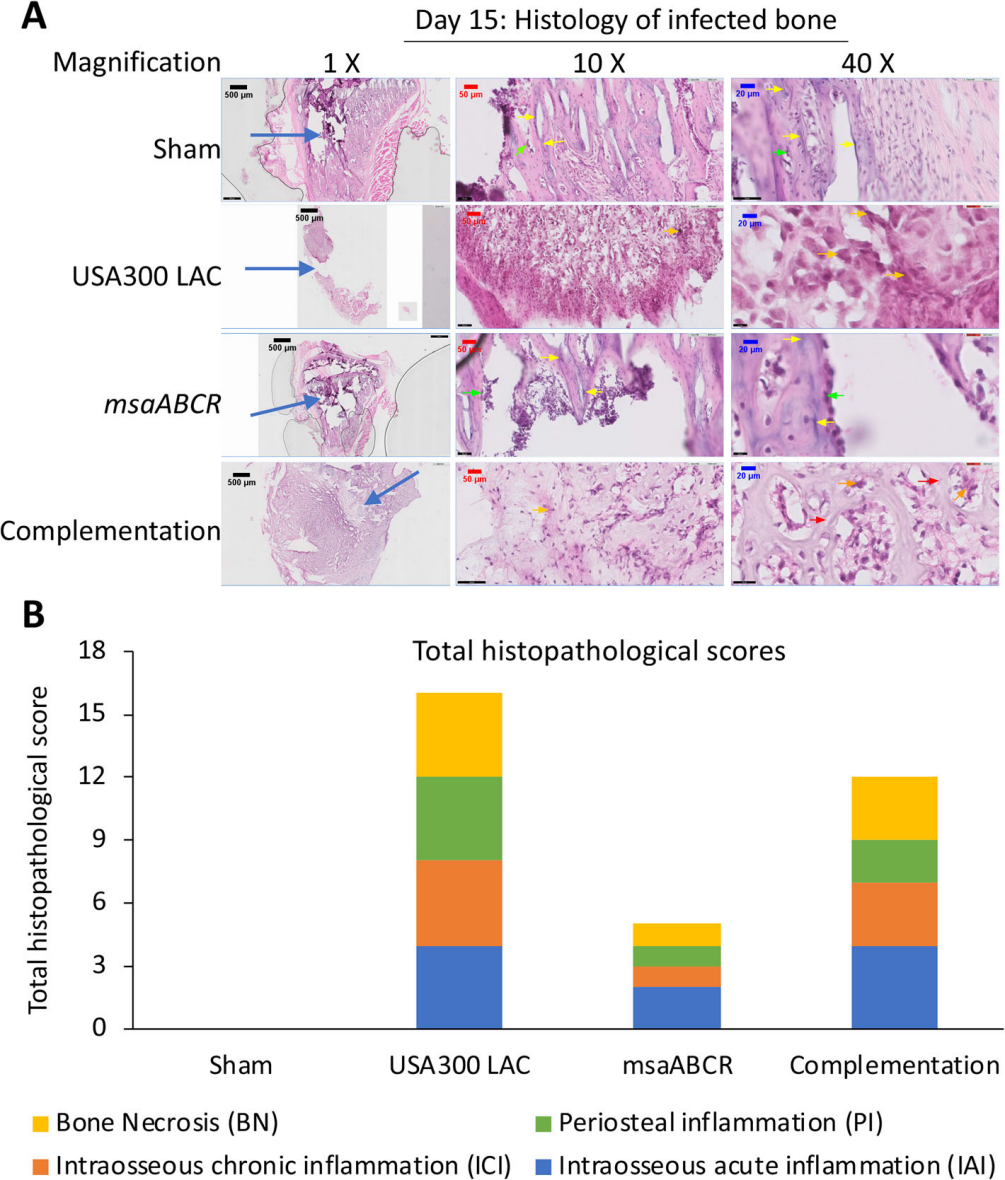
Hematoxylin and eosin (H&E) stained sections of representative bone samples after day 15 post-infection. Bone infected with sham pins (control) or with the USA300 LAC, *msaABCR mutant*, or complementation strains (**a**) at low (left) and high (right) magnification. Blue arrows show the points of insertion of the K-wires, green arrows show osteoblast cells, red arrows show osteoclasts cells, yellow arrows show newly formed bones, and orange arrows show bone necrosis. Total histopathological scores of infected bone (**b**). Histopathological scores were assessed on the basis of intraosseous acute inflammation (IAI, blue), intraosseous chronic inflammation (ICI, orange), periosteal inflammation (PI, green), and bone necrosis (BN, yellow)

Histopathological analysis of bone infected with sham group after day 15 showed an active bone healing process, with no evidence of bone necrosis or acute and chronic intraosseous and periosteal inflammation (Fig. 8a). The hallmark feature of bone infected with the USA300 LAC strain is the presence of large multinucleated osteoclasts and severe bone necrosis with multiple foci of sequestra formation (Fig. 8a). Moreover, we also observed severe chronic intraosseous inflammation, intramedullary abscess, and fibrosis with subperiosteal abscess formation in the USA300 LAC infected bone sample (Fig. 8a). Bone infected with *msaABCR* mutant showed mild intraosseous and periosteal inflammation. However, subperiosteal abscess, intramedullary abscess and fibrosis were not observed in the bone infected with *msaABCR* mutant. Although, a single focus of bone necrosis without any sequestrum formation was observed, active chondrocyte activity with newly formed bones were also observed in the *msaABCR* infected bone (Fig. 8a). Bone infected with the complementation strain also showed severe intraosseous inflammation with intramedullary abscess, mild chronic inflammation with significant intramedullary fibrosis, and severe chronic inflammation without any subperiosteal abscess formation (Fig. 8a). Severe bone necrosis with a single focus of sequestrum was also observed in the complementation strain infected bone (Fig. 8a). Overall, sham bone received a total histopathological score of 0, whereas, USA300 LAC, *msaABCR* mutant, and complementation strains received total scores of 16, 5, and 12 respectively (Fig. 8b).

### Host immunological response against staphylococcal infection

**Fig. 9:**
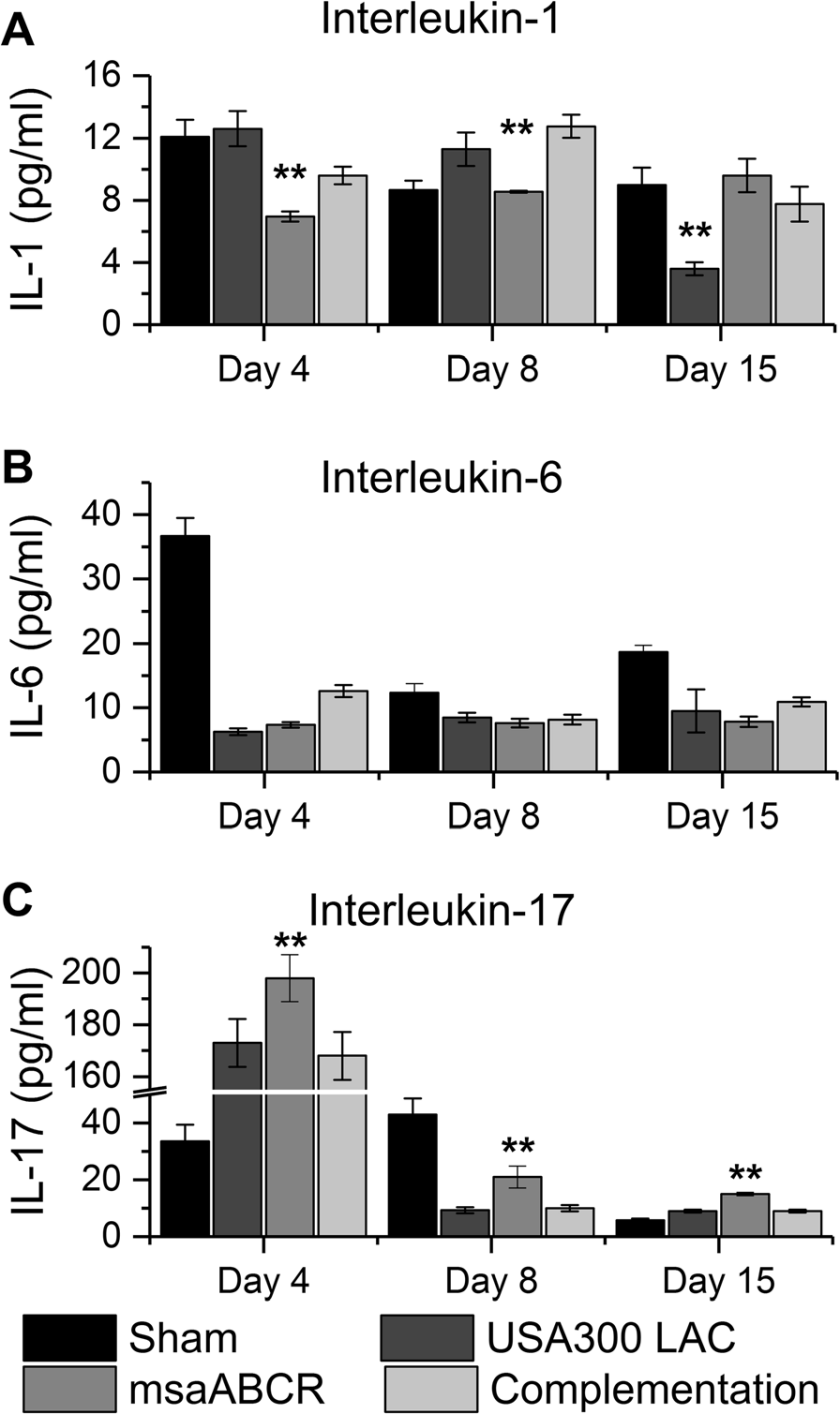
Immune response against staphylococcal bone infection. Blood samples were collected after each infection period (on days 4, 8 and 15) and were processed to obtain serum. Quantitative measurements of IL-1 (**a**), IL-17 (**b**), and IL-6 (**c**) were performed. All values were analyzed using one-way ANOVA followed by a post-hoc Tukey test. Error bars indicate standard errors of the means. A *P*-value of < 0.05 was considered statistically significant (**p-value < 0.01)

In this study we focused on three main pro-inflammatory cytokines (IL-1, IL-6, and IL-17) that have been previously linked to staphylococcal OM infections. We observed a significant decrease in IL-1 serum levels in animals infected with *msaABCR* mutant relative to USA300 LAC and complementation strains after day 4 and day 8 post-infection. However, after day 15 post-infection, IL-1 serum levels in the animals infected with USA300 LAC strain was significantly less compared to the animals infected with *msaABCR* mutant, complementation strains, and the animals that received sham K-wires (Fig. 9a). At this moment we have no clear picture about reduced IL-1 levels in the blood samples infected with USA300 LAC strains after day 15 post-infection. We did not observe any significant differences in the IL-6 serum levels in all test strains during all post-infection periods (Fig. 9b). However, the IL-6 response in the sham group was significantly higher than in all three test groups (Fig. 9b). The animals infected with *msaABCR* mutant showed significant increase in IL-17 levels relative to the animals infected with the USA300 LAC and complementation strains during all post-infection periods (Fig. 9c).

### The *msaABCR* mutant group showed increased ALP activity

**Fig. 10:**
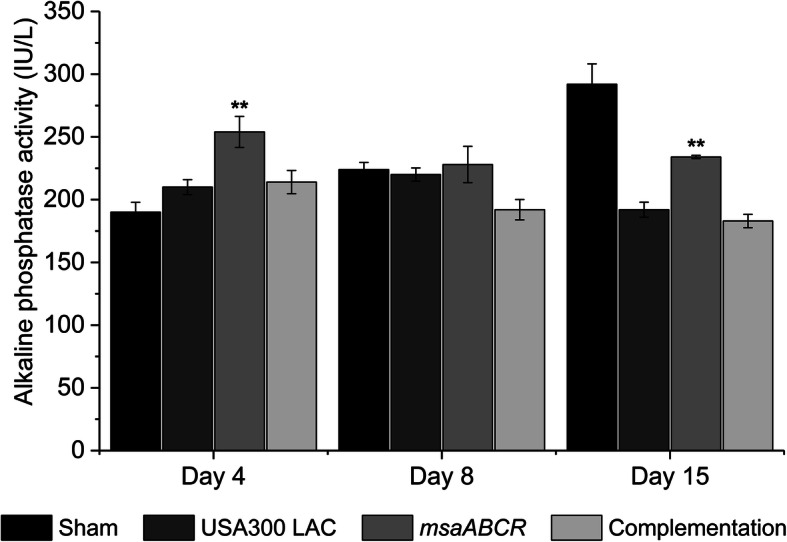
Alkaline phosphatase (ALP) activity measurements in serum. Alkaline phosphatase activity in serum was measured after each infection period (on days 4, 8, and 15) as an indicator of bone metabolic activity. All values were analyzed using one-way ANOVA followed by a post-hoc Tukey test. Error bars indicate standard errors of the means. A *P*-value of < 0.05 was considered statistically significant (**p-value < 0.01)

In this study, we measured ALP from the post-mortem blood samples as a marker of bone reformation. Our results showed significant increase in ALP activity in the animals infected with the *msaABCR* mutant relative to the animal group infected with USA300 LAC and complementation strains on day 4 and day 15 post-infection (Fig. 10). However, on day 8 post-infection, we observed no statistically significant difference in ALP activity between the test strains (Fig. 10).

### The *msaABCR* proteome suggests defects in proteins important for staphylococcal OM

Previously, we showed that deletion of the *msaABCR* operon affected the expression of 238 genes that play roles in metabolism, virulence, and biofilm development. *msaABCR* transcriptomics also showed that several genes and operons that play roles in pathogenesis (*fnbA, lukF-PV, lukS-PV, sarA,* the *splABCDEF* operon, and the *sspABC* operon) were affected [33]. In previous studies these genes and operons have been implicated in staphylococcal OM. In this study whole-cell proteomics of the *msaABCR* mutant was performed and compared with the USA300 LAC strain under planktonic and biofilm growth conditions. Whole-cell proteomics of the *msaABCR* mutant under planktonic growth conditions revealed that a total of 187 proteins were differentially expressed relative to the USA300 LAC strain (Additional file 3: Table S1), whereas under biofilm conditions 170 proteins were differentially expressed (Additional file 4: Table S2).

Deletion of the *msaABCR* operon led to decreased expression of cell adhesion factors, virulence factors, proteases, and immunomodulators that are important for colonizing bone, interacting with osteoblasts / osteoclasts, modulates secreted virulence factors and evade host immune system (Table 2). Under planktonic growth conditions, *msaABCR* proteomes showed reduced production of cell adhesion factors such as Fibronectin binding protein A (FnbA), clumping factor A (ClfA), clumping factor B (ClfB), serine–aspartate repeat-containing protein D (SdrD), and Extracellular matrix binding protein (Embp), but showed increased production of serine–aspartate repeat-containing protein C (SdrC) (Table 2). Under biofilm growth conditions, only clumping factor A (clfA) was significantly downregulated, whereas fibronectin-binding protein A (FnbA), fibronectin-binding protein B (FnbB), serine–aspartate repeat-containing protein C (SdrC), serine–aspartate repeat-containing protein D (SdrD), and Extracellular matrix binding protein (Embp) were upregulated (Table 2).

**Table 2:** Proteins involved in progression and pathogenicity of staphylococcal osteomyelitis infection

Proteins that interacts with bone matrix and osteoblasts cells						
MSCRAMM(s)	Functional response	Mean peptide spectral count				References
		Planktonic condition		Biofilm condition		
		USA300 LAC	*msaABCR* mutant	USA300 LAC	*msaABCR* mutant	
Fibronectin binding protein A (FnbA)	Internalization	2	0	81	128	[42, 43]
Fibronectin binding protein B (FnbB)	Internalization	–	–	**0**	11*	[42, 43]
Serine-aspartate repeat-containing proteins (SdrC)	Colonization	**0**	8*	–	–	[43]
Serine-aspartate repeat-containing proteins (SdrD)	Colonization	**36**	12*	0	2	[43]
Extracellular matrix binding protein (Embp)	Colonization	**7**	2*	**0**	3*	[44]
**Toxins and exoproteins involved in osteoblast cytotoxicity and induce osteoclastogenesis**						
**Toxins / exoproteins**						
Alpha hemolysin (Hla)	Osteoblast/osteoclast cell death	**19**	6*	–	–	[45, 46]
Panton-Valentine leucocidin (PVL)	Persistence of infection	37	24	203	308	[20, 21, 47, 48]
Uncharacterized leukocidin-like protein 1 (LukL1)	Osteoblast cytotoxicity	**215**	83*	232	332	[20, 48]
Staphylococcal protein A (SpA)	Induction of bone loss (apoptosis) and bone destruction (osteoclastogenesis), inhibits mineralization	–	–	**12**	5*	[47, 49, 50]
**Other virulence factors that plays role in osteomyelitis infection and immune evasion**						
**Virulence factors**						
Serine protease (sspA)	modulates secreted osteolytic factors	**0**	38*	**–**	**–**	[46, 51–53]
Cysteine protease (SspB)		**12**	66*	**–**	**–**	
Cysteine protease (SspC)		**0**	12*	**7**	22*	
Aureolysin (Aur)		**0**	60*	**–**	**–**	
Staphopain A	immunomodulatory protein that blocks neutrophil recruitment	37	31	**0**	5*	[54]
Map protein	Immunomodulators interfering T cell mediated response	**85**	0*	**116**	0*	[55]
Immunoglobulin-binding protein sbi	Immune evasion factor	**46**	19*	98	114	[56]
Clumping factor A (ClfA)	Unknown	11	6	**16**	5*	[57]
Clumping factor B (ClfB)	Unknown	**9**	3*	33	44	[57]

Proteins differentially expressed ≥2-fold is considered significant (*) and is shown in bold

Staphylococcal virulence factors such as alpha-hemolysin (Hla) and uncharacterized leucocidin-like protein 1 (LukL1), which leads to osteoblast death or triggers osteoclastogenesis and promotes persistence of infection, were downregulated significantly in the *msaABCR* mutant under planktonic conditions (Table 2). Under biofilm growth condition staphylococcal protein A (SpA) was downregulated (Table 2). Extracellular proteases, such as aureolysin, serine proteases, cysteine proteases, and thermonucleases, are produced at a significantly higher level by the *msaABCR* mutant in planktonic growth conditions. However, under biofilm growth conditions extracellular proteases such as cysteine protease and staphopain A are produced at a significantly higher level (Table 2).

Staphylococcal immune modulator that has been previously studied to interfere with T-cell mediated host immune system, MAP protein, was undetectable in *msaABCR* proteomics under both planktonic and biofilm growth condition (Table 2). Staphylococcal immune evasion factor, Immunoglobulin-binding protein (Sbi), was significantly downregulated under planktonic growth condition (Table 2). In addition, several proteins that play roles in metabolism, DNA replication, transcription and translation, transport systems, and pathogenesis were also affected (Additional file 4: Tables S1 and Additional file 5: Table S2).

## Discussion

Community-associated MRSA USA300 LAC strain is causing increasing numbers of OM cases in the US, with approximately 100,000 new cases every year [7, 22, 23, 58]. When OM infections are associated with implants and biofilm, they are very difficult to treat and often lead to life-threatening complications such as septicemia, venous thrombosis, and other pathologic fracture conditions [59–61]. During recent years the common use of implant devices in all surgical fields has caused a drastic increase in the number of implants and a higher risk of implant-associated infections. In this study we applied a similar surgical procedure to study implant-associated biofilm OM models using a rat model as previously described that closely mimic the clinical OM infections [39, 40]. We evaluated end-point quantification of viable cells from K-wires and infected bone samples using clinical isolates of CA-MRSA strain USA300 LAC, *msaABCR* mutant, and *msaABCR* complementation strains. We showed that biofilm formation on the inserted K-wire implant and the number of intraosseous bacterial CFUs in the bone samples were significantly reduced in animals infected with the *msaABCR* mutant compared with animals infected with the USA300 LAC and complementation strains during all post-infection periods. We also showed that the extent of bone damage and/or loss in the *msaABCR* mutant infected bone was significantly reduced accompanied by increased osteoblast response and evidenced the reactive bone formation. Whereas, USA300 LAC and complementation strains showed significant bone damage and/or loss, accompanied by massive osteoclast activation, severe bone necrosis with multiple foci of sequestra formation, and with chronic intramedullary abscess and intramedullary fibrosis. Therefore, this study showed that the infection of bone with USA300 LAC strains resulted in an imbalance of activity between osteoclast and osteoblast activity and triggered massive bone damage, whereas the impact was minimal when *msaABCR* operon was mutated. Thus, we conclude that the *msaABCR* operon indeed plays a role in implant-associated OM infection in a rat model.

Previous studies have shown the role of proinflammatory cytokines and chemokines in maintaining the balance between bone resorption and formation by recruiting and activating infiltrated immune cells during bacterial infection of bone [62–66]. Host cytokines such as IL-1, IL-6, and IL-17 are important markers of bone metabolism under pathological conditions [63, 65, 66]. IL-1 is a potent cytokine that is involved in bone resorption by inducing osteoclast differentiation at inflammatory sites during bone infection [62, 63]. The response of the T_H_ 17-associated cytokines IL-6 and IL-17 has been shown to play an important role in bacterial infection in human *S. aureus*-associated OM and in animal models [64, 65, 67]. IL-17A promotes bone formation by stimulating osteoblastic proliferation and differentiation, which facilitates bone fracture healing [65]. Another study has shown that an increased IL-6 response activates osteoclast differentiation, thereby stimulating increased bone resorption activity in an osteoblast cell culture model [64]. In this study, we showed that infection with USA300 LAC triggers IL-1 production but represses IL-17 production, thus leading to increased osteoclast activity. Whereas the *msaABCR* mutant infected animals showed reduced IL-1 level and increased IL-17 level, thus supporting increased osteoblast response, and reactive bone formation as demonstrated by microCT and histopatholgical analysis of infected bone. While we have not tested other host factors that trigger bone degradation and/or formation in response to *S. aureus* infection, our result showed that *msaABCR* mutant is defective in stimulating the host immune response to trigger osteoclast activation, which mediates bone degradation. We also observed significantly reduced IL-1 serum levels in the animals infected with USA300 LAC strain compared to the animals infected with *msaABCR* mutant, complementation strains, and the animals that received sham K-wires at day 15 post-infection. This is in contrast with histopathological data that showed large multinucleated osteoclasts activity and severe bone necrosis in the USA300 LAC infected bone relative to *msaABCR* mutant infected bone. At this moment we have no clear picture about reduced IL-1 levels in the blood samples infected with USA300 LAC strains after day 15 post-infection. In addition, we also observed increased IL-6 response in sham group compared to all test group. This result is in contrast with previous result [64] that showed an increased IL-6 response in both human and mouse osteoblasts when infected with *S. aureus*; however, this difference might be due to a difference in serum level response (this study) versus the osteoblast local response in the previous study.

Bone-specific ALP is synthesized by osteoblasts in response to bone injury and plays a role in bone matrix calcification [67]. In studies on long bone fracture healing, alkaline phosphatase was shown to correlate with fracture callus volume, bone formation, and fracture-healing rates [68–70]. We observed significantly increased ALP activity in the animals infected with *msaABCR* mutant. This observation correlates with the microCT data and bone histopathological data concerning the infected bone, in which the animals that received a sham pin and those infected with the *msaABCR* mutant showed increase osteoblast response during all post-infection periods and evidence of newly formed bones after day 15 post-infection. Whereas the reduced ALP activity in animals infected with the wild type and complementation strains may be the result of increase osteoclast and less osteoblast activity, increased bone loss, and with no evidence of any reactive bone formation even after 15 days post-infection.

Several studies showed that specific staphylococcal proteins (MSCRAMMS and virulence factors) play a role during the initial interaction with bone and the progression of OM infection [12, 42, 43, 47, 49, 50, 67, 71–73]. Bone extracellular matrix and bone cells are important targets for microbes, especially Staphylococci, once the bone is exposed to the external environment [6, 12, 74]. In a situation in which medical implants or K-wires are inserted into the bone, the tissue–implant surface can be a substrate for the attachment of the host extracellular matrix proteins fibronectin, fibrinogen, albumin, vitronectin, and collagen. The host matrix protein-coated K-wires provide a niche for the attachment of hosts cells, such as fibroblasts, osteoblasts, and endothelial cells, but also facilitate the attachment of bacteria such as *S. aureus,* thus promoting bacterial colonization and biofilm formation [12, 44, 75–79]. Studies have shown that staphylococcal clones that possess *cna*, *bbp*, and/or *fnbB* genes are efficient in attaching to bone extracellular matrix and concentrating around the osteoblasts. Of all the *S. aureus* strains that cause OM, 10–60% express *cna*, *bbp*, and *fnbB*, whereas all *S. aureus* strains express either *fnbA* or *clumping factors A* and *B* [57, 80, 81]. *S. aureus* also produces several virulence factors that can directly interact with osteoblasts and bone extracellular matrix, thus triggering increased ostecloclastogenesis and causing serious bone damage in the host [12, 20, 21, 42, 47–50, 61, 67, 71–74, 77]. Crémieux et al. [48] showed that Panton-Valentine leucocidin (PVL), produced by CA-MRSA strains, induces increased bone deformation and muscular abscess in an OM study in a rabbit model. In addition, CA-MRSA strains of *S. aureus* also overexpress several other virulence factors, such as α-hemolysin (Hla), phenol-soluble modulin (psms), and proteases, which are critical virulence factors in several animal models for brain abscesses, skin and soft-tissue infections, and pneumonia [45, 82, 83]. Loughran et al. [73] studied acute, posttraumatic OM using a murine model and compared the extent of virulence in two clinical isolates of staphylococcal strains (USA300 LAC and USA200 UAMS-1). They found that the USA300 LAC strain showed increased virulence and bone damage compared with the UAMS-1 strain, although the difference was not statistically significant. They also found increased osteolysis by a CA-MRSA strain of *S. aureus* (USA300 LAC), which was partly due to its ability to express high levels of accessory gene regulator (agr), resulting in the production of high levels of extracellular toxins, such as phenol-soluble modulins (PSMs) and alpha toxins, which were shown to be more cytotoxic to osteoblasts in vitro. In this study we showed that several staphylococcal proteins that facilitates *S. aureus* to attach bone extracellular matrix components, infect bone osteoblasts, and modulates host immune system were significantly affected in the *msaABCR* mutant (Table 2). Several staphylococcal virulence factors that are cytotoxic to osteoblasts, triggers osteoclastogenesis, and induces bone loss and/or destruction are also affected in the *msaABCR* mutant (Table 2). Several previous studies have linked increased protease production with defective biofilm formation, reduced accumulation of secreted virulence factors, reduced toxicity to osteoblasts and osteoclasts cells in vitro, and reduced virulence and attenuation in OM infection [31, 46, 51–53, 73, 84]. Cassat et al. 2013 [46] showed that extracellular-secreted proteases, mainly aureolysin (Aur), significantly reduced the abundance of staphylococcal exoproteomes that are important for host-tissue binding, biofilm formation, invasion of host cells, and the cytolytic factors that trigger cortical bone destruction. Increased extracellular proteases (SspA, SspB, SspC, and Aur) that leads to increased cell death and defective biofilm formation was one of the major phenotypes of *msaABCR* mutant [31, 32]. In this study, *msaABCR* whole cell proteomics analysis also showed significant production of SspA, SspB, SspC, Aur, and staphopain A. A study by Rom JS et al., [84] showed reduced accumulation of alpha toxin (Hla) and extracellular protein A in the *msaABCR* mutant [84]. They also showed that deletion of *msaABCR* leads to the significant decrease in accumulation of extracellular proteins [84]. Findings from this study and study by Rom JS et al., [84] suggest that decreased pathogenicity and/or decreased bone destruction by the *msaABCR* mutant may be mainly due to increased proteases production and/or activity, thus affecting the abundance of extracellular proteins required for host-tissue binding (cell adhesion factors), hosts cells invasion, evasion of immune system, and osteolytic factors such as LukA and alpha hemolysins that have been shown to play role in osteomyelitis in previous studies [20, 46, 48, 54–56]. However, the *msaABCR* proteomics were performed in in vitro conditions and not from in-vivo samples. Therefore, additional studies are needed to further define the contribution of various *msaABCR*-regulated molecules to pathogenesis during osteomyelitis infection.

## Conclusions

In this study we showed that the *msaABCR* mutant is defective in several cell-surface adhesion protein factors that are important for interacting with and colonizing host cells, such as osteoblasts, and host cell matrix, including bone extracellular matrix. In addition, the *msaABCR* mutant is also defective in some important virulence factors, such as hemolysin and leukocidins, which can kill host cells, such as osteoblasts, and their dissemination that can lead to infection progression. Thus, the *msaABCR* operon mutant is defective in implant-associated in vivo biofilm formation on K-wires, and decreased intraosseous survivability, that led to reduced bone damage and therefore it plays a role in implant-associated chronic OM in rat model. The findings from this study provide additional insights into the pathogenesis of implant-associated chronic OM infections by USA300 LAC and explore the potential for targeting *msaABCR* for therapy. It should be however noted that while we observed reactive bone formation in the *msaABCR* mutant similarly to the sham controls, the *msaABCR* mutant still caused some degree of bone destruction. This implicates other virulence factors, whose production were not impacted by the deletion of *msaABCR* operon and should be investigated further to fully understand osteomyelitis pathogenesis caused by *S. aureus*.

## Methods

### Bacterial strains and growth media

Overnight bacterial cultures (the USA300 LAC strain, the *msaABCR* operon deletion mutant (*msaABCR* mutant) strain [32], and a complementation strain [32] were prepared by inoculating cells from frozen culture stocks into culture tubes containing 5 mL of freshly prepared tryptic soy broth (TSB) and incubating at 37 °C with continuous shaking at 225 rpm. The overnight cultures were diluted 1:10 in fresh medium (TSB or biofilm medium: TSB supplemented with 3% sodium chloride and 0.5% glucose) and incubated for 2 h to prepare a starter culture for further experiments. The starter culture was normalized to an optical density at 600 nm (OD600) of 0.05 (2X10^7^ CFUs per ml) in prewarmed TSB for planktonic growth conditions and in prewarmed biofilm media for biofilm growth conditions.

### Sprague Dawley rats and experimental design

This study included 96 animals randomly divided using a random number generator into 4 groups (*n* = 24) USA300 LAC, *msaABCR* mutant, complementation, and sham. All animals underwent a surgical procedure to place a K-wire with or without bacteria, and three animals per group were sacrificed immediately following the surgical procedure to assess bacterial load. The remaining 21 animals per group (7 animals per time point based upon power analysis) were euthanized on days 4, 8, and 15 by an overdose of 5% isoflurane (2-chloro-2-(difluoromethoxy)-1,1,1-trifluoro-ethane) followed by bilateral pneumothorax prior to collection of blood, vital organs, and bone. Post-mortem blood samples were processed to obtain plasma and serum, aliquoted into storage tubes, and stored at − 80 °C. Five legs were placed on ice and processed for bacterial counts, while 2 legs were processed for microCT analysis of bone followed by histopathological analysis. Vital organs (heart, spleen, kidneys, and lungs) were collected and immediately frozen at − 80 °C for analysis of bacterial load resulting from dissemination from the infection site. Organ samples for histopathological studies were placed in buffered formalin.

### An implant-associated OM model using the Sprague Dawley rat (SD-rat)

The surgical procedure was performed as previously described for an animal model of chronic OM, with slight modifications [39, 40]. In brief, the sterile K-wires (0.035″ × 7″ TROCAR-TROCAR, Jorgensen Laboratories Inc., Loveland, CO) were aseptically cut in half (0.035″ × 3.5″) and coated with SD-RAT plasma overnight at 4 °C to facilitate colonization with *S. aureus* cells. Biofilm formation on the K-wires was initiated by incubating them at 37 °C for 24 h with the test strains (USA300 LAC, *msaABCR* mutant, and complementation) pre-normalized to an OD_600_ of 0.05 (2X10^7^ CFUs per ml). The biofilm-coated K-wires were stored at − 80 °C until use and for not more than 7 days. Six biofilm-coated K-wires were used to enumerate the bacterial CFUs prior to the surgical procedure to evaluate the number of initial bacterial CFUs. Sterile K-wires coated with SD-Rat plasma were used as negative control (sham infection). The K-wires colonized with test strains were inserted into the bones of Sprague Dawley rats (SD-Rat) to induce OM.

Following approval from the University of Mississippi Medical Center institutional Animal Care and Use Committee (IACUC), a total of 96 SD-rats weighing 250–300 g were purchased from Charles River Labs. The animals were housed under a 12-h day/night light cycle and allowed access to food and water ad libitum. The animals were acclimated for 1 week prior to surgical manipulation. The animals were randomly divided using a random number generator into into 3 sets of 4 groups (*n* = 24), and a K-wire was inserted surgically. Isoflurane (2%) was used for anesthesia induction followed by intraperitoneal injection of ketamine (75 mg/kg) and xylazine (5 mg/kg) for surgical anesthesia, and a presurgical dose of Buprenorphine (Temgesic, Schering-Plough, Brussels, Belgium) (0.05 mg/Kg) for analgesia. The skin above the bone was disinfected using 70% ethanol, providone iodine scrub, providone, followed by 70% ethanol. Following the aseptic surgical preparation, the position of the bone metaphysis was determined by palpation, and the K-wire pin was implanted transcortically through the metaphysis. Afterwards, the pin was bent on both ends and cut adjacent to the skin to secure it in the bone. Six hours post-surgery, the animals received a second subcutaneous injection of 0.10 mg/kg buprenorphine (Temgesic, Schering-Plough, Brussels, Belgium). Following surgery, the animals were housed individually for the first 3 days post-surgery, provided Buprenorphine (0.1 mg/kg) for pain every 12 h during the first 2 days and then assessed for pain daily, and analgesics were provided as needed. Wounds were evaluated for infection daily for the duration of the experiment. Animal cages were cleaned daily for the first 3 days, then every other day for the remainder of the experiment. The bedding was collected into biohazard bags, and the cages disinfected before entering the cage wash area.

### Bacterial counts

To determine the number of bacterial inoculums or colony-forming units (CFUs) prior to infection, the K wires coated with each bacterial strain (3 animals per group) were inserted in rat bone. All the animals were euthanized immediately, and K-wires and bone samples were harvested to enumerate the bacterial CFUs retained in the bone and on the K-wires after surgery.

#### Bone samples

Infected bone samples (5 per group) with K-wire pins were carefully excised from the bone, and the bone shaft was removed using a scalpel. The bone was cut into smaller sections, homogenized in the presence of liquid nitrogen using mortar and pestle. The samples were suspended in 5 ml of 1X PBS, and plated in tryptic soy agar (TSA) agar plates after serial dilutions in sterile 1x PBS to determine the viable bacterial count, as previously described [39].

#### K-wire samples

The K-wires (5 per group) were carefully removed from the infected bone samples and rinsed twice with sterile 1x PBS. The K-wires were suspended in 5 ml of sterile 1x PBS and sonicated for 30 min in a 2.8-L ultrasonic bath (Fischer Scientific) to release the bacteria from the biofilms that had formed on the pins. Appropriate serial dilutions were performed and plated on TSA media to count the total number of bacterial CFUs present on the K-wire samples.

#### Blood samples

The blood samples were collected from all animals for enumeration of bacterial CFUs (7 animals per group). One hundred microliters of blood samples were serially diluted in 1 ml of 1X PBS and plated on TSA media to count the total number of bacterial CFUs present in the blood samples after overnight incubation at 37 °C.

#### Organ samples

Six organ samples per group (heart, spleen, liver, kidney, and lung) were collected in a sterile plastic container and immediately stored at –80 °C until use. They were later retrieved from the − 80 °C freezer and rinsed with sterile 1x PBS thrice. The organ samples were cut into small pieces using a sterile surgical blade and transferred to a 15-ml FastPrep tube containing 5 ml of an equal mixture of 0.5-mm and 1-mm zirconia/silica beads (BioSpec Products). Five milliliters of sterile 1x PBS was added, and the beads beating was performed in FastPrep-24 instrument (4 times, with a setting of 6.5 M/S for 60 s and 2 min off time on ice). The cell debris was removed by a quick spin at 2000 x g for 2 min, and the supernatant was transferred to another 15-ml tube. The samples were serially diluted and plated on TSA media to count the total number of bacterial CFUs present in the organ samples after overnight incubation at 37 °C.

### Micro-computed tomography (microCT) analysis of infected bone

High-resolution analysis of cortical thickness, cortical bone destruction, and underlying trabecular bone in the sham group and the bacteria-treated groups was determined by microCT imaging using a 1172 μCT SkyScan scanner with a 10-megapixel camera (Skyscan, Aartselaar, Belgium) and the manufacturer’s analytical software. The SkyScan scanning parameters were set up as previously described by Tucci et al. [85]. Structural indices were calculated using CTAn software (Skyscan, Aartselaar, Belgium) and BoneJ software [86]. Differences in cortical bone destruction were analyzed using one-way ANOVA followed by a post-hoc Tukey test. A *P*-value of < 0.05 was considered statistically significant.

### Histopathological analysis of infected bone

For histopathological analysis, samples of the proximal half of the bone were fixed in neutral-buffered formalin solution, decalcified using Cal-Ex™ solution, embedded in OCT for frozen sectioning, and cut into 10-μm sections. The sections were stained with hematoxylin and eosin (H & E). Histopathological scores were determined for intraosseous acute inflammation (IAI), intraosseous chronic inflammation (ICI), periosteal inflammation (PI), and bone necrosis (BN) using the system previously described by Smeltzer et al. [41]. Each parameter was scored on a 5-point scale (0–4), and the scores for each section were added together to provide a total histopathological score, as shown in Table 1.

### ELISA testing for IL-6, IL-1, and IL-17

Commercially available colorimetric sandwich enzyme-linked immunosorbent (ELISA) assays were used to obtain quantitative measurements of IL-1, IL-17, and IL-6 (R&D Systems). The manufacturer’s protocols were followed to determine the concentrations of cytokines in the sample. Briefly, either 25 μL of standard or plasma sample was dispensed in duplicate into microtiter plate wells coated with IL-6, IL-17, or IL-1. The plate was incubated for 2 h to allow the cytokine present in the sample or standard to bind to the immobilized antibody. The plates were washed with 100 μL of the wash buffer supplied by the manufacturer three times, and then 25 μL of a second biotinylated cytokine antibody was added and the plate incubated overnight at 4 °C. Following the overnight incubation, the plate was washed three additional times with 100 μL of wash buffer per wash. After washing, the unbound biotinylated antibody, which was conjugated to HRP with streptavidin, was pipetted into the wells, and incubated for 1 h. The wells were washed again in a similar manner, and 50 μL of TMB substrate solution were added to the wells for 20 min followed by the addition of 50 μL of stop solution. The intensity of the color in the samples was compared with a standard curve generated by the color intensity of the concentration standards at 405 nm, and the amount of cytokine in the samples was thereby determined.

### Alkaline phosphatase (ALP) assay

The activity of ALP in serum was determined by measuring the rate of hydrolysis of various phosphate esters under specified conditions. In the Pointe Scientific (Canton, MI) kinetic assay, p-nitrophenyl phosphate is hydrolyzed to p-nitrophenol and inorganic phosphate and detected at 405 nm, and the rate of hydrolysis is directly proportional to ALP activity. Working reagents were prepared according to the manual instructions. One milliliter of reagent was pipetted into a spectrophotometer cuvette and warmed to 37 °C for 5 min. The reference cuvette contained water and was used as the blank. A plasma aliquot of 0.25 mL was added to warm reagent and allowed to incubate at 37 °C for an additional minute. The absorbance was determined at 405 nm, and the cuvette was then placed back in the 37 °C heat block and measured again after 5 and 10 min. The average absorbance difference per minute was calculated and multiplied by a factor of 2187 to convert the results to IU/L.

### Proteomics: analysis of the *Staphylococcus aureus* proteome using a label-free strategy

For whole cell proteomics analysis, the cells were grown to OD600 of 1.5 in TSB broth for planktonic growth condition. The cells were collected, washed twice with ice cold 1X PBS and were used for whole cell proteomics analysis. For biofilm growth condition, the biofilm was grown in flow cells (Stovall Life Science, Greensboro, NC). The flow cells were pre-coated with human plasma. One hundred microliter of starter bacterial suspension were inoculated into the flow cells by injection and allowed to incubate at 37 °C for 1 h. Biofilm media was then pumped through the flow cells at a flow rate of 0.5 ml/min for 24 h. The biofilm cells were collected, washed twice with ice cold 1X PBS and were used for whole cell proteomics analysis.

#### Trypsin digestion

Protein extraction was performed using urea buffer (8 M urea, 150 mM NaCl, 50 mM Tris-HCl, pH 8) and mechanical disruption with 1.0-mm silica beads in a Bullet Blender (Next Advance). Samples were centrifuged at 10,000 x g for 10 min and the supernatant removed. The protein concentration was determined using a Qubit fluorometry assay (Invitrogen). Protein concentrations were normalized with lysis buffer. Five hundred micrograms of protein from each sample was reduced with 10 mM dithiothretiol for 30 min at room temperature and alkylated with 10 mM iodoacetamide for 45 min at room temperature. Trypsin was added at a 1:30 enzyme-to-substrate ratio, and urea was diluted to 1.6 M with 25 mM ammonium bicarbonate. The reaction was terminated with 0.1% trifluoroacetic acid (TFA) and the suspension clarified by centrifugation at 10,000 x g for 10 min.

#### Solid-phase extraction (SPE)

Cleared peptide solution was processed by SPE on a Waters HLB PRiME with 30-mg-capacity cartridges using 5 psi. In brief, samples were loaded directly onto the cartridge per the vendor protocol and washed with 2 × 1 mL of 0.1% TFA. Peptides were eluted with 2 × 500 μL of 90% acetonitrile with 0.1% TFA, and the peptide concentrations were determined by UV absorbance at 280 nm.

#### Mass spectrometry

Two micrograms of each sample were analyzed by nano-LC-MS/MS (MSBioworks, Ann Arbor, MI, USA) with a Waters NanoAcquity HPLC system interfaced to a ThermoFisher Q Exactive mass spectometer using a 4-h reverse-phase gradient. Peptides (30 μL) were loaded on a trapping column and eluted over a 75-μm analytical column at 350 nL/min, and both columns were packed with Jupiter Proteo resin (Phenomenex, Torrance, CA, USA). The mass spectrometer was operated, and the data was obtained in data dependent mode with the Orbitrap operating settings as previously described by Franco M et al. [87].

#### Data processing

A composite database comprising the 20 sequenced *S. aureus* strains was created. The strains were 04–02981, bovine RF122 (ET3–1), COL, ED98, JH1, JH9, JKD6159, MRSA ST398 (isolate S0385), MRSA252, MSSA476, Mu3 (ATCC 700698), Mu50 (ATCC 700699), MW2, N315, NCTC 8325, Newman, TW20 (0582), USA300 (TCH1516), and ED133. Data were analyzed using Mascot software with the parameter settings as previously described by Franco M et al. [87]. Mascot DAT files were parsed for processing with Scaffold software for validation, filtering, and to create a nonredundant list per sample. Data were filtered using a 1% protein and peptide false discovery rate (FDR) and required at least one unique peptide per protein.

### Comparative gene ontology analysis of proteomics data

As a part of the functional genomic analysis of our whole-cell proteomics data, we performed gene ontology (GO) classification of differentially expressed (≥3-fold) proteins, as previously described [33]. We used a freely available web-based GO analysis tool (Comparative GO, [88, 89]) to better understand the differentially expressed proteins in terms of the biological pathways involved.

### Statistical analysis

All statistical analyses to test for significance in this study were done using OriginPro software (Originlab, Northampton, MA). A significance level of 0.05 was set as the cutoff value while performing statistical analyses. All the data obtained from CFU experiments were analyzed using one-way ANOVA followed by a post-hoc Tukey test.

## Supplementary information

**Additional file 1: Fig. S1.** Microbial counts from vital organ samples to study staphylococcal dissemination. The organ samples (*n* = 6): liver (**a**), kidney (**b**), and lung (**c**) from sham and infected animals were harvested after each infection period (on days 4, 8, and 15) and were processed to enumerate the bacterial CFUs, as described in methods. The difference in microbial count between the USA300 LAC and *msaABCR mutant* strains was analyzed using one-way ANOVA followed by a post-hoc Tukey test. Error bars indicate standard errors of the means. A *P*-value of < 0.05 was considered statistically significant.

**Additional file 2: Fig. S2.** Structural indices of sham and infected bones after day 4 post-infection**.** Analysis of bone trabecular microarchitecture (percentage bone volume, BV/TV; bone surface/volume ratio, BS/BV; trabecular pattern factor, Tb.Pf; trabecular thickness, Tb.Th; trabecular spacing, Tb.Sp; and trabecular number, Tb.N) were performed using Nrecon and BoneJ software. All values were analyzed using one-way ANOVA followed by a post-hoc Tukey test. Error bars indicate standard errors of the means. A P-value of < 0.05 was considered statistically significant (**p-value < 0.01).

**Additional file 3: Fig. S3.** Structural indices of sham and infected bones after day 8 post-infection. Analysis of trabecular bone microarchitecture (percentage bone volume, BV/TV; bone surface/volume ratio, BS/BV; trabecular pattern factor, Tb.Pf; trabecular thickness, Tb.Th; trabecular spacing, Tb.Sp; and trabecular number, Tb.N) were performed Nrecon and BoneJ software. All values were analyzed using one-way ANOVA followed by a post-hoc Tukey test. Error bars indicate standard errors of the means. A *P*-value of < 0.05 was considered statistically significant (**p-value < 0.01).

**Additional file 4: Table S1.** Comparative Gene Enrichment analysis of *msaABCR* proteomics under planktonic conditions.

**Additional file 5: Table S2.** Comparative gene enrichment analysis of *msaABCR* proteomes under biofilm growth condition.

## Data Availability

All data generated or analyzed during this study are included in this published article [and its supplementary information files].

## Abbreviations

CA-MRSA: community-associated methicillin-resistant *S. aureus*;
SD-Rat: Sprague Dawley Rat
OM: Osteomyelitis
μCT: microCT
CFUs: Colony forming units
°C: Degree Celsius
TSB: Tryptic soy broth
Biofilm medium: TSB supplemented with 3% sodium chloride and 0.5% glucose
H&E: Hematoxylin and eosin
IAI: Intraosseous acute inflammation
ICI: Intraosseous chronic inflammation
PI: Periosteal inflammation
BN: Bone necrosis (BN)
IL: Interleukin
BV/TV: percent bone volume
BS/BV: bone surface/volume ratio
Tb.Pf: trabecular pattern factor
Tb.th: trabecular thickness
Tb.N: trabecular number
TB.Sp: trabecular separation
HRP: Horseradish peroxidase
TMB: Tetramethylbenzidine
ALP: Alkaline phosphatase
IU/L: International Units Per Litre
SPE: Solid phase extraction
TFA: trifluoroacetic acid
GO: Gene ontology
mg/kg: milligram per kilogram
PBS: Phosphate buffer saline
ml: milliliter
mm: milliliter
μl: microliter
