# Supplementary material for: The role of the msaABCR operon in implant-associated chronic osteomyelitis in Staphylococcus aureus USA300 LAC
Source: BMC Microbiol. 2020 Oct 27;20:324. doi: 10.1186/s12866-020-01964-8 (PMC7590495; doi:10.1186/s12866-020-01964-8)
Supplement: Supplementary file 4 — Additional file 4: Table S1. Comparative Gene Enrichment analysis of msaABCR proteomics under planktonic conditions. [file 12866_2020_1964_MOESM4_ESM.docx]

**Additional File 4: Table S1: Comparative Gene Enrichment analysis of *msaABCR* proteomics under planktonic conditions**

| **Aminoacid metabolism** | | | | | | | | | | |  |
| --- | --- | --- | --- | --- | --- | --- | --- | --- | --- | --- | --- |
| **Aminoacid Metabolism** | | **Gene ID** | | **Protein ID** | | **Protein name** | **USA300 LAC** | | ***msaABCR* mutant** | |  |
| Glutamate biosynthetic process | | SAUSA300_0445 | | A0A0H2XEH4_STAA3 | | Glutamate synthase, large subunit | 4 | | 12 | |  |
| Biosynthesis of threonine, isoleucine, and methionine via homoserine intermediate | | SAUSA300_1226 | | A0A0H2XHR4_STAA3 | | Homoserine dehydrogenase | 9 | | 23 | |  |
| Glycine, serine and threonine metabolism via L-homoserine | | SAUSA300_1228 (thrB) | | KHSE_STAA3 | | Homoserine kinase | 0 | | 5 | |  |
| Lysine biosynthetic process via diaminopimelate biosynthetic process | | SAUSA300_1289 (dapA) | | DAPB_STAA3 | | 4-hydroxy-tetrahydrodipicolinate reductase (HTPA reductase) | 2 | | 8 | |  |
|  | | SAUSA300_1290 (dapB) | | DAPH_STAA3 | | 2,3,4,5-tetrahydropyridine-2,6-dicarboxylate N-acetyltransferase | 8 | | 27 | |  |
| Isoleucine, valine biosynthetic process | | SAUSA300_2009 (ilvC) | | ILVC_STAA3 | | Ketol-acid reductoisomerase (NADP(+)) | 2 | | 13 | |  |
| Leucine biosynthetic process | | SAUSA300_2010 (leuA) | | LEU1_STAA3 | | 2-isopropylmalate synthase | 0 | | 4 | |  |
| Glycine biosynthetic process from serine | | SAUSA300_2067 (glyA) | | GLYA_STAA3 | | Serine hydroxymethyltransferase | 32 | | 79 | |  |
| Alanyl-tRNA aminoacylation | | SAUSA300_1575 (alaS) | | SYA_STAA3 | | Alanine--tRNA ligase | 0 | | 31 | |  |
| Arginine biosynthetic process | | SAUSA300_0864 (argG) | | ASSY_STAA3 | | Argininosuccinate synthase (Citrulline--aspartate ligase) | 2 | | 5 | |  |
| Nitrogen compound metabolic process | | SAUSA300_1987 | | A0A0H2XHP6_STAA3 | | Hydrolase, carbon-nitrogen family | 3 | | 12 | |  |
| Urea catabolic process | | SAUSA300_2243 (ureG) | | UREG_STAA3 | | Urease accessory protein UreG | 3 | | 9 | |  |
|  | | SAUSA300_2240 (ureC) | | URE1_STAA3 | | Urease subunit alpha (Urea amidohydrolase subunit alpha) | 0 | | 3 | |  |
| L-alanine catabolic process | | SAUSA300_1331 (ald1) | | DHA1_STAA3 | | Alanine dehydrogenase 1 | 61 | | 16 | |  |
| L-threonine catabolic process to propionate | | SAUSA300_1330 (tdcB) | | TDCB_STAA3 | | L-threonine dehydratase catabolic TdcB | 24 | | 6 | |  |
| **Carbohydrate metabolism** | | | | | | | | | | |  |
| Cellular aldehyde metabolic process | | SAUSA300_1901 (aldA2) | | A0A0H2XFU7_STAA3 | | Aldehyde dehydrogenase | 6 | | 15 | |  |
| N-acetylglucosamine metabolic process | | SAUSA300_0554 (nagB) | | NAGB_STAA3 | | Glucosamine-6-phosphate deaminase | 12 | | 3 | |  |
| Alcohol metabolic process | | SAUSA300_0151 (adhE) | | A0A0H2XG10_STAA3 | | Aldehyde-alcohol dehydrogenase | 90 | | 22 | |  |
| Mannitol metabolic process | | SAUSA300_2108 (mtlD) | | MTLD_STAA3 | | Mannitol-1-phosphate 5-dehydrogenase | 23 | | 6 | |  |
| **Lipid Metabolism** | | | | | | | | | | |  |
| Phospholipid biosynthetic process | | SAUSA300_1176 (pgsA) | | A0A0H2XHR7_STAA3 | | CDP-diacylglycerol--glycerol-3-phosphate 3-phosphatidyltransferase | 5 | | 0 | |  |
|  |  | SAUSA300_0711 | | Y711_STAA3 | | Putative lipid kinase | 16 | | 6 | |  |
| Glycerol metabolic process | | SAUSA300_0637 | | A0A0H2XFB7_STAA3 | | Dihydroxyacetone kinase, DhaL subunit | 5 | | 2 | |  |
|  |  | SAUSA300_0636 | | A0A0H2XGF3_STAA3 | | Dihydroxyacetone kinase, DhaK subunit | 5 | | 2 | |  |
| Steroid biosynthetic process | | SAUSA300_0329 | | A0A0H2XG46_STAA3 | | Putative oxidoreductase | 0 | | 3 | |  |
| Teichoic acid biosynthetic process | | SAUSA300_0628 | | A0A0H2XED9_STAA3 | | Teichoic acid biosynthesis protein D | 0 | | 3 | |  |
| **DNA and RNA metabolism** | | | | | | | | | | |  |
| rRNA processing | | SAUSA300_0981 | | A0A0H2XDU9_STAA3 | | class I SAM-dependent rRNA methyltransferase | 7 | | 0 | |  |
|  | | SAUSA300_0469 (rnmV) | | A0A0H2XGL4_STAA3 | | Ribonuclease M5 | 4 | | 0 | |  |
| Nucleotide catabolic process | | SAUSA300_0025 | | A0A0H2XI02_STAA3 | | 5'-nucleotidase family protein | 5 | | 0 | |  |
| DNA metabolic process | | SAUSA300_1960 | | A0A0H2XGX3_STAA3 | | Putative phage-related DNA recombination protein | 5 | | 0 | |  |
| RNA catabolic process | | SAUSA300_2352 | | A0A0H2XJ67_STAA3 | | Addiction module toxin, Txe/YoeB family | 0 | | 7 | |  |
| Purine nucleoside metabolic process | | SAUSA300_2091 (deoD) | | A0A0H2XEF6_STAA3 | | Purine nucleoside phosphorylase DeoD-type (PNP) | 0 | | 3 | |  |
| **Vitamin biosynthesis** | | | | | | | | | | |  |
| Mo-molybdopterin cofactor biosynthetic process | | SAUSA300_2225 (moaC) | | MOAC_STAA3 | | Cyclic pyranopterin monophosphate synthase | 0 | | 4 | |  |
| Menaquinone biosynthetic process | | SAUSA300_1737 (menE) | | MENE_STAA3 | | 2-succinylbenzoate--CoA ligase | 0 | | 5 | |  |
| Chorismate biosynthetic process | | SAUSA300_1357 (aroC) | | AROC_STAA3 | | Chorismate synthase | 15 | | 37 | |  |
| Shikimate metabolic process | | SAUSA300_1555 (aroE) | | AROE_STAA3 | | Shikimate dehydrogenase (NADP(+)) (SDH) | 3 | | 8 | |  |
| Tetrahydrofolate interconversion | | SAUSA300_0965 (folD) | | FOLD_STAA3 | | Bifunctional protein FolD | 0 | | 34 | |  |
| Biosynthetic process | | SAUSA300_2497 | | A0A0H2XFY9_STAA3 | | Aminotransferase | 0 | | 5 | |  |
| Coenzyme A biosynthetic process | | SAUSA300_2084 (coaW) | | COAW_STAA3 | | Type II pantothenate kinase | 4 | | 0 | |  |
| 7,8-dihydroneopterin 3'-triphosphate biosynthetic process | | SAUSA300_0551 (folE2) | | GCH4_STAA3 | | GTP cyclohydrolase FolE2 | 14 | | 0 | |  |
|  | |  | |  | |  |  | |  | |  |
| **Transport** | | | | | | | | | | |  |
| Transport | | SAUSA300_0888 (oppC) | | A0A0H2XEU4_STAA3 | | Oligopeptide ABC transporter, permease protein | 0 | | 4 | |  |
|  | | SAUSA300_2176 (ecfA1) | | ECFA1_STAA3 | | Energy-coupling factor transporter ATP-binding protein EcfA1 (ECF transporter A component EcfA1) | 0 | | 3 | |  |
|  | | SAUSA300_0625 (tagG) | | A0A0H2XIF1_STAA3 | | Oligopeptide ABC transporter, permease protein | 3 | | 0 | |  |
| Transmembrane transport | | SAUSA300_0145 | | A0A0H2XKE4_STAA3 | | Phosphonate ABC transporter | 0 | | 5 | |  |
| Intracellular protein transmembrane transport | | SAUSA300_2584 (secA2) | | SECA2_STAA3 | | Protein translocase subunit SecA 2 | 0 | | 4 | |  |
| Potassium ion transport | | SAUSA300_0911 | | A0A0H2XH32_STAA3 | | Transporter, monovalent cation:proton antiporter-2 (CPA2) family protein | 0 | | 4 | |  |
| Methionine transport | | SAUSA300_0435 (metN1) | | METN1_STAA3 | | Methionine import ATP-binding protein | 0 | | 8 | |  |
| Peptide transport | | SAUSA300_0889 (oppD) | | A0A0H2XJ10_STAA3 | | Oligopeptide ABC transporter, ATP-binding protein | 2 | | 5 | |  |
| Sodium ion transport | | SAUSA300_0676 | | A0A0H2XFJ2_STAA3 | | Anion transporter family protein | 3 | | 0 | |  |
| Transporter activity | | SAUSA300_2213 | | A0A0H2XER4_STAA3 | | AcrB/AcrD/AcrF family protein | 24 | | 0 | |  |
|  | | SAUSA300_0115 (sirC) | | A0A0H2XEX4_STAA3 | | Iron compound ABC transporter, permease protein SirC | 4 | | 0 | |  |
|  | | SAUSA300_0332 | | A0A0H2XFR4_STAA3 | | PTS system, IIA component | 5 | | 0 | |  |
|  | | SAUSA300_0352 | | A0A0H2XH88_STAA3 | | ABC transporter, ATP-binding protein | 8 | | 3 | |  |
|  | | SAUSA300_2358 | | A0A0H2XGB6_STAA3 | | ABC transporter, permease protein | 2 | | 5 | |  |
|  | | SAUSA300_0704 | | A0A0H2XIP2_STAA3 | | ABC transporter, ATP-binding protein | 4 | | 12 | |  |
|  | | SAUSA300_0706 | | A0A0H2XET0_STAA3 | | Putative osmoprotectant ABC transporter, ATP-binding protein | 3 | | 8 | |  |
| Amino acid transmembrane transporter activity | | SAUSA300_0566 | | A0A0H2XKI8_STAA3 | | APC family permease | 5 | | 0 | |  |
| Ion channel activity | | SAUSA300_1244 (mscL) | | MSCL_STAA3 | | Large-conductance mechanosensitive channel | 2 | | 23 | |  |
| ATP synthesis coupled electron transport | | SAUSA300_0855 (mnhA1) | | MNHA1_STAA3 | | Na(+)/H(+) antiporter subunit A1 (Mnh complex subunit A1) | 3 | | 0 | |  |
|  | | SAUSA300_2399 | | A0A0H2XE61_STAA3 | | ABC transporter, ATP-binding protein | 4 | | 0 | |  |
|  | | SAUSA300_0285 | | ESXB_STAA3 | | ESAT-6 secretion system extracellular protein B (Ess extracellular protein B) | 0 | | 3 | |  |
| Quaternary-ammonium-compound-transporting ATPase activity | | SAUSA300_2392 (opuC) | | A0A0H2XKJ8_STAA3 | | Glycine betaine/carnitine/choline ABC transporter permease | 6 | | 0 | |  |
|  | |  | |  | |  |  | |  | |  |
| **DNA replication, transcription, and translation** | | | | | | | | | | |  |
| DNA replication | | SAUSA300_1346 (dinG) | | DING_STAA3 | | Probable ATP-dependent helicase DinG homolog | 2 | | 6 | |  |
| DNA replication, synthesis of RNA primer | | SAUSA300_1105 (priA) | | A0A0H2XFV2_STAA3 | | Primosomal protein N' (ATP-dependent helicase PriA) | 3 | | 0 | |  |
| Regulation of DNA repair | | SAUSA300_1854 (recX) | | RECX_STAA3 | | Regulatory protein RecX | 3 | | 0 | |  |
| DNA repair | | SAUSA300_0004 (recF) | | RECF_STAA3 | | DNA replication and repair protein RecF | 0 | | 4 | |  |
|  | | SAUSA300_1243 (sbcC) | | SBCC_STAA3 | | Nuclease SbcCD subunit C | 10 | | 3 | |  |
| Chromosome segregation | | SAUSA300_1447 (xerD) | | A0A0H2XE73_STAA3 | | Tyrosine recombinase XerD | 0 | | 3 | |  |
| Double-strand break repair via homologous recombination | | SAUSA300_0869 (addB) | | ADDB_STAA3 | | ATP-dependent helicase/deoxyribonuclease subunit B (AddB) | 2 | | 5 | |  |
| DNA recombination | | SAUSA300_1598 (ruvA) | | RUVA_STAA3 | | Holliday junction ATP-dependent DNA helicase RuvA | 3 | | 0 | |  |
| Nucleotide-excision repair | | SAUSA300_1045 (uvrC) | | UVRC_STAA3 | | UvrABC system protein C (Protein UvrC) | 3 | | 0 | |  |
| Base-excision repair | | SAUSA300_0563 (ung) | | UNG_STAA3 | | Uracil-DNA glycosylase (UDG) | 0 | | 3 | |  |
| DNA restriction-modification system | | SAUSA300_0196 (hsdR) | | A0A0H2XKA8_STAA3 | | Type I restriction enzyme R Protein | 9 | | 23 | |  |
| DNA primase and helicase activity | | SAUSA300_1522 (dnaG) | | A0A0H2XHU2_STAA3 | | DNA primase | 0 | | 6 | |  |
| Transcription, DNA-templated | | SAUSA300_1220 | | A0A0H2XGQ9_STAA3 | | DNA-binding response regulator, LuxR family | 0 | | 4 | |  |
|  | | SAUSA300_1992 (agrA) | | A0A0H2XH08_STAA3 | | Accessory gene regulator protein A | 10 | | 3 | |  |
|  | | SAUSA300_0683 | | A0A0H2XH29_STAA3 | | Transcriptional regulator, DeoR family | 6 | | 2 | |  |
|  | | SAUSA300_1347 (birA) | | A0A0H2XG87_STAA3 | | Bifunctional ligase/repressor BirA | 3 | | 0 | |  |
|  | | SAUSA300_0137 | | A0A0H2XK05_STAA3 | | Transcriptional regulator, GntR family | 3 | | 0 | |  |
|  | | SAUSA300_2480 | | A0A0H2XI75_STAA3 | | Transcriptional regulator, LysR family | 0 | | 3 | |  |
| Regulation of transcription, DNA-templated | | SAUSA300_1455 | | A0A0H2XJB0_STAA3 | | Transcriptional regulator, AraC family | 0 | | 3 | |  |
|  | | SAUSA300_2106 | | A0A0H2XI57_STAA3 | | Putative transcriptional regulator | 4 | | 0 | |  |
|  | | SAUSA300_0777 | | A0A0H2XII8_STAA3 | | Cold shock protein | 38 | | 144 | |  |
| Regulation of transcription, DNA-templated | | SAUSA300_1455 | | A0A0H2XJB0_STAA3 | | Transcriptional regulator, AraC family | 0 | | 3 | |  |
|  | | SAUSA300_2106 | | A0A0H2XI57_STAA3 | | Putative transcriptional regulator | 4 | | 0 | |  |
|  | | SAUSA300_0777 | | A0A0H2XII8_STAA3 | | Cold shock protein | 38 | | 144 | |  |
| Translation | | SAUSA300_0366 (rpsF) | | RS6_STAA3 | | 30S ribosomal protein S6 | 0 | | 60 | |  |
| tRNA pseudouridine synthesis | | SAUSA300_2173 (truA) | | TRUA_STAA3 | | tRNA pseudouridine synthase A | 3 | | 0 | |  |
| tRNA wobble adenosine to inosine editing | | SAUSA300_0543 (tadA) | | A0A0H2XFN7_STAA3 | | tRNA-specific adenosine deaminase | 0 | | 3 | |  |
|  | |  | |  | |  |  | |  | |  |
| **Pathogenesis** | | | | | | | | | | |  |
| Plasminogen activation | | SAUSA300_1922 (saK) | | A0A0H2XFS8_STAA3 | | Staphylokinase | 0 | | 4 | |  |
| Cell adhesion | | SAUSA300_0546 (sdrC) | | SDRC_STAA3 | | Serine-aspartate repeat-containing protein C | 0 | | 8 | |  |
|  | | SAUSA300_2565 (clfB) | | A0A0H2XHK2_STAA3 | | Clumping factor B | 9 | | 3 | |  |
|  | | SAUSA300_0547 (sdrD) | | SDRD_STAA3 | | Serine-aspartate repeat-containing protein D | 36 | | 12 | |  |
|  | | SAUSA300_RS10495 (map-w) | | A0A0H3JY83_STAAW | | Truncated map-w protein | 85 | | 0 | |  |
| Virulence factors | | SAUSA300_1974 | | LUKL1_STAA3 | | Uncharacterized leukocidin-like protein 1 (LukL1) | 215 | | 83 | |  |
|  | | SAUSA300_1058 | | A0A0H2XEW5_STAA3 | | Alpha-hemolysin | 19 | | 6 | |  |
|  | | SAUSA300_2364 (sbi) | | SBI_STAA3 | | Immunoglobulin-binding protein sbi | 46 | | 19 | |  |
|  | | SAUSA300_1327 (embp) | | EBH_STAA3 | | Extracellular matrix-binding protein embp | 7 | | 2 | |  |
|  | | SAUSA300_0800 (sek) | | A0A0H2XJE8_STAA3 | | Staphylococcal enterotoxin K | 5 | | 2 | |  |
|  | | SAUSA300_0283 | | A0A0H2XEV3_STAA3 | | EssC protein | 6 | | 19 | |  |
|  | | SAUSA300_2589 (sraP) | | SRAP_STAA3 | | Serine-rich adhesin for platelets (Staphylococcus aureus surface protein A) | 5 | | 16 | |  |
|  | | SAUSA300_2253 | | A0A0H2XFI7_STAA3 | | Secretory antigen SsaA | 0 | | 3 | |  |
|  | | SAUSA300_0899 | | MECA_STAA3 | | Adapter protein MecA | 0 | | 4 | |  |
|  | | SAUSA300_1514 (fur) | | A0A0H2XHQ6_STAA3 | | Ferric uptake regulation protein | 3 | | 9 | |  |
|  | | SAUSA300_1029 (isdA) | | ISDA_STAA3 | | Iron-regulated surface determinant protein A | 0 | | 4 | |  |
| Metalloendopeptidase | | SAUSA300_2572 (aur) | | A0A0H2XDQ5_STAA3 | | Zinc metalloproteinase aureolysin | 0 | | 60 | |  |
| Serine-type endopeptidase | | SAUSA300_0951 (sspA) | | A0A0H2XFC3_STAA3 | | Serine protease | 0 | | 38 | |  |
| Cysteine-type endopeptidase inhibitor | | SAUSA300_0949 (sspC) | | A0A0H2XE71_STAA3 | | Cysteine protease | 0 | | 12 | |  |
| Cysteine-type peptidase | | SAUSA300_0950 (sspB) | | A0A0H2XGH9_STAA3 | | Cysteine protease | 12 | | 66 | |  |
| Nuclease | | SAUSA300_1222 (nuc) | | A0A0H2XGE3_STAA3 | | Thermonuclease | 4 | | 0 | |  |
| SOS response | | SAUSA300_0741 (uvrB) | | A0A0H2XGZ8_STAA3 | | UvrABC system protein B (Protein UvrB) (Excinuclease ABC subunit B) | 16 | | 6 | |  |
|  | | SAUSA300_1045 (uvrC) | | UVRC_STAA3 | | UvrABC system protein C (Protein UvrC) (Excinuclease ABC subunit C) | 3 | | 0 | |  |
|  | | SAUSA300_0004 (recF) | | RECF_STAA3 | | DNA replication and repair protein RecF | 0 | | 4 | |  |
| Phosphorelay signal transduction system | | SAUSA300_1638 (phoR) | | A0A0H2XFS9_STAA3 | | Sensory box histidine kinase PhoR | 4 | | 0 | |  |
|  | | SAUSA300_1866 (vraS) | | A0A0H2XJC3_STAA3 | | Sensor protein VraS | 0 | | 3 | |  |
|  | | SAUSA300_1220 | | A0A0H2XGQ9_STAA3 | | DNA-binding response regulator, LuxR family | 0 | | 4 | |  |
|  | | SAUSA300_1992 (agrA) | | A0A0H2XH08_STAA3 | | Accessory gene regulator protein A | 0 | | 4 | |  |
|  | |  | |  | |  |  | |  | |  |
| **Others** | | | | | | | | | | |  |
| Pentose-phosphate shunt, non-oxidative branch | SAUSA300_2283 (rpiA) | | A0A0H2XDX1_STAA3 | | Ribose-5-phosphate isomerase A | | | 10 | | 4 | |
| Tricarboxylic acid cycle | SAUSA300_2312 (mqo) | | A0A0H2XIW4_STAA3 | | Probable malate:quinone oxidoreductase | | | 8 | | 3 | |
| Aminoacylase activity | SAUSA300_2276 | | A0A0H2XG69_STAA3 | | Peptidase, M20/M25/M40 family | | | 0 | | 3 | |
| ferredoxin-NADP+ reductase activity | SAUSA300_2319 | | FENR_STAA3 | | Ferredoxin--NADP reductase (FNR) (Fd-NADP(+) reductase) | | | 0 | | 3 | |
| Lactoyl-glutathione lyase activity | SAUSA300_1088 | | A0A0H2XEH6_STAA3 | | Glyoxalase family protein | | | 0 | | 3 | |
| protein kinase activity | SAUSA300_0509 (mcsB) | | MCSB_STAA3 | | Protein-arginine kinase | | | 3 | | 10 | |
| N-acetyltransferase activity | SAUSA300_0591 | | A0A0H2XEU1_STAA3 | | Acetyltransferase, GNAT family | | | 3 | | 0 | |
|  | SAUSA300_1312 | | A0A0H2XFM8_STAA3 | | Acetyltransferase, GNAT family | | | 12 | | 4 | |
|  | SAUSA300_2316 | | A0A0H2XGV3_STAA3 | | Acetyltransferase, GNAT family | | | 8 | | 3 | |
|  | SAUSA300_0943 | | A0A0H2XJX3_STAA3 | | Acetyltransferase, GNAT family family | | | 0 | | 4 | |
|  | SAUSA300_0745 | | A0A0H2XH79_STAA3 | | Putative acetyltransferase | | | 4 | | 0 | |
|  | SAUSA300_0053 | | A0A0H2XGJ0_STAA3 | | Spermidine N(1)-acetyltransferase | | | 0 | | 3 | |
| Transaminase activity | SAUSA300_1579 | | A0A0H2XKI8_STAA3 | | Aminotransferase, class V | | | 0 | | 3 | |
|  | SAUSA300_0381 (nfrA) | | NFRA_STAA3 | | NADPH-dependent oxidoreductase | | | 0 | | 8 | |
| Transferase activity, transferring glycosyl groups | SAUSA300_0549 | | A0A0H2XI10_STAA3 | | Glycosyl transferase, group 1 family protein | | | 3 | | 11 | |
| Hydrolase activity | SAUSA300_1051 | | A0A0H2XFL3_STAA3 | | Phosphoesterase | | | 0 | | 5 | |
|  | SAUSA300_0557 | | A0A0H2XIY3_STAA3 | | HAD-superfamily hydrolase, subfamily IA, variant 1 | | | 0 | | 4 | |
|  | SAUSA300_1291 | | Y1291_STAA3 | | Uncharacterized hydrolase | | | 0 | | 3 | |
| tRNA dihydrouridine synthase activity | SAUSA300_0089 | | A0A0H2XGZ4_STAA3 | | tRNA-dihydrouridine synthase | | | 8 | | 3 | |
| Carboxylic ester hydrolase activity | SAUSA300_0763 (st) | | A0A0H2XJL0_STAA3 | | Carboxylesterase | | | 0 | | 8 | |
| rRNA (pseudouridine-N3-)-methyltransferase activity | SAUSA300_0026 (rlmH) | | RLMH_STAA3 | | Ribosomal RNA large subunit methyltransferase H | | | 2 | | 5 | |
| rRNA (guanine-N7-)-methyltransferase activity | SAUSA300_2644 (rsmG) | | RSMG_STAA3 | | Ribosomal RNA small subunit methyltransferase G | | | 4 | | 0 | |
| protein lipoylation | SAUSA300_0744 (lgt) | | LGT_STAA3 | | Prolipoprotein diacylglyceryl transferase | | | 0 | | 4 | |
| butanediol metabolic process | SAUSA300_2166 (alsS) | | A0A0H2XHS9_STAA3 | | Alpha-acetolactate synthase | | | 0 | | 6 | |
| FtsZ-dependent cytokinesis | SAUSA300_1074 (ftsL) | | A0A0H2XH39_STAA3 | | Cell division protein FtsL | | | 5 | | 0 | |
| acetoin biosynthetic process | SAUSA300_2536 (budA) | | A0A0H2XFQ6_STAA3 | | Alpha-acetolactate decarboxylase | | | 0 | | 4 | |
|  |  | |  | |  | | |  | |  | |
| **Other Uncharacterized Proteins** | | | | | | | | | | | |
|  | SAUSA300_0753 | | Y753_STAA3 | | Epimerase family protein | | | 12 | | 4 | |
|  | SAUSA300_1938 | | A0A0H2XEL4_STAA3 | | Phi77 ORF006-like protein, putative capsid protein | | | 3 | | 0 | |
|  | SAUSA300_0474 | | A0A0H2XK95_STAA3 | | Putative endoribonuclease L-PSP | | | 3 | | 9 | |
|  | SAUSA300_0325 | | A0A0H2XG03_STAA3 | | Glycine cleavage H-protein | | | 3 | | 0 | |
|  | SAUSA300_0783 | | A0A0H2XI45_STAA3 | | Phosphoglycerate mutase family protein | | | 0 | | 3 | |
|  | SAUSA300_0070 | | A0A0H2XHZ1_STAA3 | | Putative lysophospholipase | | | 18 | | 4 | |
|  | SAUSA300_2614 | | A0A0H2XEH9_STAA3 | | Putative lipoprotein | | | 0 | | 3 | |
|  | SAUSA300_0377 | | A0A0H2XHE2_STAA3 | | Putative lipoprotein | | | 18 | | 0 | |
|  | SAUSA300_2101 | | A0A0H2XJ77_STAA3 | | SAP domain protein | | | 9 | | 0 | |
|  | SAUSA300_1577 | | A0A0H2XJ15_STAA3 | | TPR domain protein | | | 5 | | 2 | |
|  | SAUSA300_1803 | | A0A0H2XIA7_STAA3 | | Uncharacterized protein | | | 7 | | 0 | |
|  | SAUSA300_0081 | | A0A0H2XHK4_STAA3 | | Uncharacterized protein | | | 5 | | 0 | |
|  | SAUSA300_0422 | | A0A0H2XEM1_STAA3 | | Uncharacterized protein | | | 5 | | 0 | |
|  | SAUSA300_1210 | | A0A0H2XEC6_STAA3 | | Uncharacterized protein | | | 4 | | 0 | |
|  | SAUSA300_0298 | | A0A0H2XIK1_STAA3 | | Uncharacterized protein | | | 4 | | 0 | |
|  | SAUSA300_2524 | | A0A0H2XI56_STAA3 | | Uncharacterized protein | | | 4 | | 0 | |
|  | SAUSA300_2212 | | A0A0H2XFZ0_STAA3 | | Uncharacterized protein | | | 3 | | 0 | |
|  | SAUSA300_0046 | | A0A0H2XF21_STAA3 | | Uncharacterized protein | | | 3 | | 0 | |
|  | SAUSA300_0031 | | A0A0H2XG12_STAA3 | | Uncharacterized protein | | | 0 | | 3 | |
|  | SAUSA300_0740 | | A0A0H2XJ54_STAA3 | | Uncharacterized protein | | | 0 | | 3 | |
|  | SAUSA300_0780 | | A0A0H2XF79_STAA3 | | Uncharacterized protein | | | 0 | | 3 | |
|  | SAUSA300_1859 | | A0A0H2XGU3_STAA3 | | Uncharacterized protein | | | 0 | | 3 | |
|  | SAUSA300_0097 | | A0A0H2XJQ0_STAA3 | | Uncharacterized protein | | | 0 | | 3 | |
|  | SAUSA300_0286 | | A0A0H2XFI6_STAA3 | | Uncharacterized protein | | | 0 | | 3 | |
|  | SAUSA300_1208 | | A0A0H2XGR3_STAA3 | | Uncharacterized protein | | | 0 | | 3 | |
|  | SAUSA300_1524 | | A0A0H2XEM9_STAA3 | | CBS domain pair protein | | | 0 | | 3 | |
|  | SAUSA300_0173 | | A0A0H2XJL5_STAA3 | | Uncharacterized protein | | | 24 | | 85 | |
|  | SAUSA300_0460 | | A0A0H2XH20_STAA3 | | Uncharacterized protein | | | 0 | | 4 | |
|  | SAUSA300_1026 | | A0A0H2XHU7_STAA3 | | Uncharacterized protein | | | 0 | | 4 | |
|  | SAUSA300_2381 | | A0A0H2XGN1_STAA3 | | Uncharacterized protein | | | 0 | | 4 | |
|  | SAUSA300_2529 | | A0A0H2XH33_STAA3 | | Uncharacterized protein | | | 0 | | 4 | |
|  | SAUSA300_1665 | | A0A0H2XIX0_STAA3 | | Uncharacterized protein | | | 3 | | 14 | |
|  | SAUSA300_1560 | | A0A0H2XIM6_STAA3 | | Uncharacterized protein | | | 0 | | 5 | |
|  | SAUSA300_1301 | | A0A0H2XEZ0_STAA3 | | Uncharacterized protein | | | 0 | | 5 | |
|  | SAUSA300_0602 | | A0A0H2XG24_STAA3 | | Uncharacterized protein | | | 35 | | 14 | |
|  | SAUSA300_1532 | | A0A0H2XGA0_STAA3 | | Uncharacterized protein | | | 10 | | 4 | |
|  | SAUSA300_1100 | | A0A0H2XGD0_STAA3 | | Uncharacterized protein | | | 3 | | 8 | |
|  | SAUSA300_1440 | | A0A0H2XET3_STAA3 | | Uncharacterized protein | | | 9 | | 24 | |
|  | SAUSA300_0174 | | A0A0H2XGQ8_STAA3 | | Uncharacterized protein | | | 0 | | 8 | |
|  | SAUSA300_2284 | | A0A0H2XFB2_STAA3 | | Uncharacterized protein | | | 0 | | 3 | |
|  | SAUSA300_2593 | | A0A0H2XEM0_STAA3 | | Uncharacterized protein | | | 3 | | 0 | |
|  | SAUSA300_0356 | | A0A0H2XFA3_STAA3 | | Uncharacterized protein | | | 2 | | 5 | |
|  | SAUSA300_0362 | | A0A0H2XJS2_STAA3 | | Uncharacterized protein | | | 0 | | 3 | |
|  | SAUSA300_2261 | | A0A0H2XI51_STAA3 | | Uncharacterized protein | | | 0 | | 3 | |
|  | SAUSA300_0084 | | A0A0H2XFN1_STAA3 | | Uncharacterized protein | | | 0 | | 3 | |
|  | SAUSA300_2004 | | A0A0H2XFJ4_STAA3 | | Uncharacterized protein | | | 0 | | 3 | |
|  | SAUSA300_0858 | | A0A0H2XF46_STAA3 | | Uncharacterized protein | | | 4 | | 3 | |
|  | SAUSA300_2209 | | A0A0H2XGP0_STAA3 | | Uncharacterized protein | | | 0 | | 3 | |
|  | SAUSA300_2593 | | A0A0H2XEM0_STAA3 | | Uncharacterized protein | | | 3 | | 0 | |
|  | SAUSA300_0843 | | A0A0H2XH04_STAA3 | | Uncharacterized protein | | | 5 | | 13 | |
|  | SAUSA300_0177 | | A0A0H2XHB0_STAA3 | | Uncharacterized protein | | | 18 | | 55 | |

Comparative GO analysis of the *msaABCR* whole cell proteomics grown under planktonic growth conditions using online tools comparative GO [88, 89]. All proteins differentially expressed ≥ 2-fold is shown.
