# Supplementary material for: The role of the msaABCR operon in implant-associated chronic osteomyelitis in Staphylococcus aureus USA300 LAC
Source: BMC Microbiol. 2020 Oct 27;20:324. doi: 10.1186/s12866-020-01964-8 (PMC7590495; doi:10.1186/s12866-020-01964-8)
Supplement: Supplementary file 5 — Additional file 5: Table S2. Comparative gene enrichment analysis of msaABCR proteomes under biofilm growth condition. [file 12866_2020_1964_MOESM5_ESM.docx]

**Additional file 5: Table S2: Comparative gene enrichment analysis of *msaABCR* proteomes under biofilm growth condition**

| **Aminoacid Metabolism** | | | | | |
| --- | --- | --- | --- | --- | --- |
| **Pathways** | **Gene ID** | **Protein ID** | **Protein name** | **USA300 LAC** | **msaABCR mutant** |
| L-alanine catabolic process | SAUSA300_1331 (ald1) | DHA1_STAA3 | Alanine dehydrogenase 1 | 0 | 157 |
| Threonine, lysine and isoleucine biosynthetic process | SAUSA300_1287 (asd) | A0A0H2XJP5_STAA3 | Aspartate-semialdehyde dehydrogenase (ASADH) | 4 | 0 |
| Glycine betaine biosynthetic process from choline | SAUSA300_2546 (betB) | A0A0H2XH11_STAA3 | Glycine betaine aldehyde dehydrogenase | 51 | 20 |
|  | SAUSA300_2545 (betA) | BETA_STAA3 | Oxygen-dependent choline dehydrogenase(CDH) | 31 | 10 |
| Glycine decarboxylation via glycine cleavage system | SAUSA300_1496 (gcvP) | GCSPB_STAA3 | Probable glycine dehydrogenase (decarboxylating) subunit 2 | 12 | 5 |
| Arginine catabolic process to ornithine | SAUSA300_0065 (arcA) | A0A0H2XGY3_STAA3 | Arginine deiminase (ADI) | 13 | 0 |
| Histidine catabolic process to glutamate and formate | SAUSA300_2281 (hutG) | HUTG_STAA3 | Formimidoylglutamase | 9 | 3 |
| L-proline biosynthetic process | SAUSA300_1452 (proC) | A0A0H2XGB3_STAA3 | Pyrroline-5-carboxylate reductase (p5C reductase) | 8 | 0 |
| 'de novo' L-methionine biosynthetic process | SAUSA300_1287 (asd) | A0A0H2XJP5_STAA3 | Aspartate-semialdehyde dehydrogenase (ASADH) | 4 | 0 |
| **Carbohydrate Metabolism** | | | | | |
| Carbohydrate metabolic process | SAUSA300_2096 (manA) | A0A0H2XHT2_STAA3 | Mannose-6-phosphate isomerase | 0 | 4 |
| L-arabinose catabolic process to xylulose 5-phosphate | SAUSA300_0537 (araB) | ARAB_STAA3 | Ribulokinase | 3 | 0 |
| **Lipid Metabolism** | | | | | |
| Fatty acid biosynthetic process | SAUSA300_2028 (acpS) | ACPS_STAA3 | Holo-[acyl-carrier-protein] synthase (Holo-ACP synthase) | 0 | 3 |
| Steroid biosynthetic process | SAUSA300_0329 | A0A0H2XG46_STAA3 | Putative oxidoreductase | 13 | 4 |
| Phospholipid biosynthetic process | SAUSA300_0711 | Y711_STAA3 | Putative lipid kinase | 4 | 0 |
| Lipid metabolic process | SAUSA300_0099 (plc) | A0A0H2XK15_STAA3 | 1-phosphatidylinositol phosphodiesterase | 2 | 7 |
| Glycolipid biosynthetic process | SAUSA300_0918 (ugtP) | UGTP_STAA3 | Processive diacylglycerol beta-glucosyltransferase | 0 | 3 |
| Glycerol metabolic process | SAUSA300_0636 | A0A0H2XGF3_STAA3 | Dihydroxyacetone kinase, DhaK subunit | 10 | 3 |
| **DNA and RNA Metabolism** | | | | | |
| 'de novo' IMP biosynthetic process | SAUSA300_0967 (purK) | A0A0H2XEX9_STAA3 | N5-carboxyaminoimidazole ribonucleotide synthase (N5-CAIR synthase) | 103 | 2 |
|  | SAUSA300_0976 (purD) | A0A0H2XFS4_STAA3 | Phosphoribosylamine--glycine ligase | 12 | 0 |
|  | SAUSA300_0972 (purF) | A0A0H2XFY4_STAA3 | Amidophosphoribosyltransferase (ATase) | 7 | 0 |
|  | SAUSA300_0969 (purS) | A0A0H2XGZ9_STAA3 | Phosphoribosylformylglycinamidine synthase subunit PurS (FGAM synthase) | 9 | 0 |
|  | SAUSA300_0973 (purM) | PUR5_STAA3 | Phosphoribosylformylglycinamidine cyclo-ligase | 7 | 0 |
|  | SAUSA300_0968 (purC) | PUR7_STAA3 | Phosphoribosylaminoimidazole-succinocarboxamide synthase | 21 | 0 |
|  | SAUSA300_0975 (purH) | PUR9_STAA3 | Bifunctional purine biosynthesis protein PurH | 12 | 0 |
|  | SAUSA300_0971 (purL) | PURL_STAA3 | Phosphoribosylformylglycinamidine synthase subunit PurL (FGAM synthase) | 31 | 2 |
|  | SAUSA300_0970 (purQ) | PURQ_STAA3 | Phosphoribosylformylglycinamidine synthase subunit PurQ (FGAM synthase) | 8 | 0 |
| 'de novo' AMP biosynthetic process | SAUSA300_0017 (purA) | A0A0H2XG07_STAA3 | Adenylosuccinate synthetase (AMPSase) | 42 | 16 |
| dTDP and dTTP biosynthetic process | SAUSA300_0459 (tmk) | KTHY_STAA3 | Thymidylate kinase (dTMP kinase) | 11 | 3 |
| Purine nucleotide metabolic process | SAUSA300_1235 (guaC) | GUAC_STAA3 | GMP reductase | 3 | 0 |
| Nucleotide catabolic process | SAUSA300_0025 | A0A0H2XI02_STAA3 | 5'-nucleotidase family protein | 14 | 4 |
| Thiamine biosynthetic process | SAUSA300_2048 (thiM) | THIM_STAA3 | Hydroxyethylthiazole kinase | 3 | 0 |
| DNA catabolic process | SAUSA300_1472 (xseA) | EX7L_STAA3 | Exodeoxyribonuclease 7 large subunit | 2 | 6 |
| **Vitamin Biosynthesis** | | | | | |
| Mo-molybdopterin cofactor biosynthetic process | SAUSA300_2225 (moaC) | MOAC_STAA3 | Cyclic pyranopterin monophosphate synthase | 2 | 5 |
| Menaquinone biosynthetic process | SAUSA300_0944 (menA) | A0A0H2XEM6_STAA3 | 1,4-dihydroxy-2-naphthoate octaprenyltransferase | 0 | 3 |
| Chorismate biosynthetic process | SAUSA300_1555 (aroE) | AROE_STAA3 | Shikimate dehydrogenase (NADP(+)) | 0 | 4 |
| Pantothenate biosynthetic process | SAUSA300_2388 (panE) | A0A0H2XJU5_STAA3 | 2-dehydropantoate 2-reductase | 0 | 3 |
| Coenzyme A biosynthetic process | SAUSA300_2084 (coaW) | COAW_STAA3 | Type II pantothenate kinase | 3 | 0 |
|  |  |  |  |  |  |
| **DNA replication, transcription and translation** | | | | | |
| DNA replication | SAUSA300_0452 (dnaX) | A0A0H2XIT2_STAA3 | DNA polymerase III subunit gamma/tau | 6 | 15 |
|  | SAUSA300_1649 (dnaE) | A0A0H2XJJ3_STAA3 | DNA-directed DNA polymerase | 2 | 5 |
|  | SAUSA300_1042 | A0A0H2XK06_STAA3 | DNA-dependent DNA polymerase family X | 2 | 8 |
|  | SAUSA300_1346 (dinG) | DING_STAA3 | Probable ATP-dependent helicase DinG homolog | 5 | 13 |
|  | SAUSA300_1242 (sbcD) | SBCD_STAA3 | Nuclease SbcCD subunit D | 0 | 3 |
|  | SAUSA300_1885 (ligA) | DNLJ_STAA3 | DNA ligase | 20 | 8 |
| Mismatch repair | SAUSA300_1188 (mutS) | A0A0H2XFL8_STAA3 | DNA mismatch repair protein MutS | 3 | 0 |
| Transcription, DNA-templated | SAUSA300_2303 (tcaR) | A0A0H2XDW4_STAA3 | Transcriptional regulator TcaR | 2 | 5 |
|  | SAUSA300_2639 | A0A0H2XH55_STAA3 | Cold shock protein | 9 | 31 |
|  | SAUSA300_0255 (lytR) | LYTR_STAA3 | Sensory transduction protein LytR | 0 | 4 |
|  | SAUSA300_2480 | A0A0H2XI75_STAA3 | Transcriptional regulator, LysR family | 3 | 0 |
|  | SAUSA300_2563 | A0A0H2XID1_STAA3 | Putative transcriptional regulator | 5 | 2 |
| Regulation of transcription, DNA-templated | SAUSA300_0777 | A0A0H2XII8_STAA3 | Cold shock protein | 69 | 285 |
|  | SAUSA300_2024 (rsbV) | A0A0H2XG98_STAA3 | Anti-sigma factor antagonist | 23 | 9 |
|  | SAUSA300_2547 | A0A0H2XFT0_STAA3 | HTH-type transcriptional regulator | 6 | 2 |
| rRNA processing | SAUSA300_0516 (mrnC) | A0A0H2XHH0_STAA3 | Mini-ribonuclease 3 | 0 | 3 |
| Translation | SAUSA300_1108 (def) | A0A0H2XGC8_STAA3 | Peptide deformylase | 2 | 5 |
|  | SAUSA300_0682 (ybaK) | A0A0H2XIY2_STAA3 | Cys-tRNA(Pro)/Cys-tRNA(Cys) | 0 | 4 |
|  | SAUSA300_1117 (rpmB) | RL28_STAA3 | 50S ribosomal protein L28 | 0 | 7 |
| tRNA thio-modification | SAUSA300_1661 (thiI) | THII_STAA3 | Probable tRNA sulfurtransferase | 2 | 7 |
| tRNA processing | SAUSA300_2070 | A0A0H2XJW1_STAA3 | Threonylcarbamoyl-AMP synthase | 2 | 5 |
| DNA binding | SAUSA300_0283 | A0A0H2XEV3_STAA3 | EssC protein | 2 | 9 |
|  | SAUSA300_1169 (ftsK) | A0A0H2XHM5_STAA3 | DNA translocase FtsK | 3 | 10 |
| Exonuclease activity | SAUSA300_1332 | A0A0H2XGQ5_STAA3 | Putative 5'-3' exonuclease | 5 | 12 |
|  |  |  |  |  |  |
| **Pathogenesis** | | | | | |
| Cell adhesion | SAUSA300_RS10495 (map-w) | A0A0E1VP30_STAA3 | MAP domain protein | 116 | 0 |
|  | SAUSA300_0772 (clfA) | A0A0H2XG16_STAA3 | Clumping factor A | 16 | 5 |
|  | SAUSA300_2440 (fnbB) | A0A0H2XKG3_STAA3 | Fibronectin binding protein B | 0 | 11 |
| Pathogenesis | SAUSA300_0113 | A0A0H2XJH7_STAA3 | Immunoglobulin G binding protein A | 12 | 5 |
|  | SAUSA300_1765 (epiC) | A0A0H2XJ01_STAA3 | Lantibiotic epidermin biosynthesis protein EpiC | 3 | 0 |
|  | SAUSA300_2142 (asp23) | ASP23_STAA3 | Alkaline shock protein 23 | 109 | 16 |
|  | SAUSA300_0407 | A0A0H2XFM7_STAA3 | Exotoxin | 0 | 7 |
|  | SAUSA300_1061 | A0A0H2XFZ1_STAA3 | Putative exotoxin 3 | 0 | 8 |
|  | SAUSA300_1068 | A0A0H2XGG8_STAA3 | Antibacterial protein | 0 | 5 |
|  | SAUSA300_0401 | A0A0H2XIH5_STAA3 | Exotoxin | 0 | 5 |
|  | SAUSA300_0403 | A0A0H2XII7_STAA3 | Exotoxin | 3 | 9 |
|  | SAUSA300_1327 (embp) | EBH_STAA3 | Extracellular matrix-binding protein embp | 0 | 3 |
|  | SAUSA300_0024 | A0A0H2XG71_STAA3 | Metallo-beta-lactamase family protein | 0 | 4 |
|  |  |  |  |  |  |
| Response to stress | SAUSA300_0067 | A0A0H2XKH6_STAA3 | Universal stress protein family | 11 | 2 |
| Response to oxidative stress | SAUSA300_0786 | OHRL_STAA3 | Organic hydroperoxide resistance protein-like | 5 | 0 |
| Carotenoid biosynthetic process | SAUSA300_2498 (crtN) | CRTN_STAA3 | Dehydrosqualene desaturase | 3 | 0 |
| Cysteine-type endopeptidase inhibitor activity | SAUSA300_0949 (sspC) | A0A0H2XE71_STAA3 | Cysteine protease | 7 | 22 |
| Cysteine-type peptidase activity | SAUSA300_1890 | A0A0H2XEH7_STAA3 | Staphopain A | 2 | 5 |
| Metallopeptidase activity | SAUSA300_1976 | A0A0H2XKA6_STAA3 | Probable succinyl-diaminopimelate desuccinylase | 3 | 0 |
| Serine-type endopeptidase activity | SAUSA300_1763 (epiP) | A0A0H2XIS6_STAA3 | Lantibiotic epidermin leader peptide processing serine protease EpiP | 9 | 0 |
| Iron uptake process | SAUSA300_1514 (furS) | A0A0H2XHQ6_STAA3 | Ferric uptake regulation protein | 0 | 3 |
| Iron assimilation | SAUSA300_1035 (isdG) | HDOX1_STAA3 | Heme oxygenase (staphylobilin-producing) 1 | 0 | 5 |
| Phosphorelay signal transduction system | SAUSA300_0255 (lytR) | LYTR_STAA3 | Sensory transduction protein LytR | 0 | 4 |
|  | SAUSA300_2558 | A0A0H2XIM9_STAA3 | Sensor histidine kinase | 0 | 3 |
|  |  |  |  |  |  |
| **Transport** | | | | | |
| Metal ion transport | SAUSA300_2495 (copZ) | COPZ_STAA3 | Copper chaperone CopZ | 6 | 0 |
| Plasma membrane ATP synthesis coupled proton transport | SAUSA300_2064 (atpB) | ATP6_STAA3 | ATP synthase subunit a | 0 | 5 |
|  | SAUSA300_2062 (atpF) | ATPF_STAA3 | ATP synthase subunit b | 20 | 8 |
| Protein transport by the Sec complex | SAUSA300_2184 (secY) | A0A0H2XIA9_STAA3 | Protein translocase subunit SecY | 0 | 4 |
| Transmembrane transport | SAUSA300_0891 (oppA) | A0A0H2XIJ5_STAA3 | Oligopeptide ABC transporter, substrate-binding protein | 4 | 0 |
|  | SAUSA300_0073 | A0A0H2XIR7_STAA3 | Peptide ABC transporter, peptide-binding protein | 152 | 0 |
|  | SAUSA300_2298 | A0A0H2XJ69_STAA3 | Multidrug resistance protein B, drug resistance transporter | 3 | 0 |
| ABC type transporter activity | SAUSA300_2213 | A0A0H2XER4_STAA3 | AcrB/AcrD/AcrF family protein | 12 | 0 |
|  | SAUSA300_1913 | A0A0H2XJV3_STAA3 | ABC transporter, ATP-binding protein | 3 | 0 |
|  | SAUSA300_2399 | A0A0H2XE61_STAA3 | ABC transporter, ATP-binding protein | 5 | 0 |
|  | SAUSA300_2358 | A0A0H2XGB6_STAA3 | ABC transporter, permease protein | 0 | 3 |
|  | SAUSA300_2453 | A0A0H2XHT0_STAA3 | ABC transporter, ATP-binding protein | 0 | 6 |
|  |  |  |  |  |  |
| **Other pathways** | | | | | |
| Gluconeogenesis | SAUSA300_2470 (sdaAB) | A0A0H2XGD2_STAA3 | L-serine dehydratase, iron-sulfur-dependent, beta subunit | 0 | 3 |
| Glycolytic process | SAUSA300_1467 (lpdA) | A0A0H2XKH9_STAA3 | Dihydrolipoyl dehydrogenase | 2 | 7 |
|  | SAUSA300_0235 (ldh1) | LDH1_STAA3 | L-lactate dehydrogenase 1(L-LDH 1) | 80 | 201 |
| Pentose-phosphate shunt | SAUSA300_1115 (rpe) | A0A0H2XGU0_STAA3 | Ribulose-phosphate 3-epimerase | 0 | 3 |
| Nitrogen compound metabolic process | SAUSA300_2244 (ureD) | URED_STAA3 | Urease accessory protein UreD | 3 | 0 |
| Cell cycle | SAUSA300_0485 | A0A0H2XFQ2_STAA3 | Cell-division initiation protein | 3 | 0 |
| Biosynthetic process | SAUSA300_0945 | A0A0H2XJA7_STAA3 | Isochorismate synthase family protein | 2 | 6 |
| Sporulation resulting in formation of a cellular spore | SAUSA300_0475 (spoVG) | SP5G_STAA3 | Putative septation protein SpoVG | 30 | 11 |
| Organic acid phosphorylation | SAUSA300_2377 | A0A0H2XHY3_STAA3 | Glycerate kinase | 4 | 0 |
| Urea catabolic process | SAUSA300_2239 (ureB) | URE2_STAA3 | Urease subunit beta | 19 | 7 |
| Acetoin biosynthetic process | SAUSA300_2536 (budA) | A0A0H2XFQ6_STAA3 | Alpha-acetolactate decarboxylase | 0 | 7 |
| Coenzyme binding | SAUSA300_2387 | A0A0H2XGM0_STAA3 | NAD dependent epimerase/dehydratase family protein | 9 | 2 |
| Acetyltransferase activity | SAUSA300_2492 | A0A0H2XI29_STAA3 | Acetyltransferase family protein | 4 | 0 |
| Aldehyde dehydrogenase (NAD) activity | SAUSA300_0170 (aldA) | ALDA_STAA3 | Putative aldehyde dehydrogenase (AldA) | 0 | 41 |
| Amino acid transmembrane transporter activity | SAUSA300_1628 (lysP) | A0A0H2XJI8_STAA3 | Lysine-specific permease | 3 | 0 |
| CDP-glycerol glycerophosphotransferase activity | SAUSA300_0626 (tagB) | A0A0H2XHY7_STAA3 | Teichoic acid biosynthesis protein B | 0 | 4 |
| Hydrolase activity | SAUSA300_2517 | A0A0H2XGH4_STAA3 | Amidohydrolase family protein | 11 | 2 |
|  | SAUSA300_0641 | A0A0H2XHH9_STAA3 | Putative lipase/esterase | 0 | 3 |
|  | SAUSA300_0557 | A0A0H2XIY3_STAA3 | HAD-superfamily hydrolase, subfamily IA, variant 1 | 0 | 4 |
| N-acetyltransferase activity | SAUSA300_0643 | A0A0H2XJQ4_STAA3 | Acetyltransferase, GNAT family | 0 | 3 |
| Nucleoside transmembrane transporter activity | SAUSA300_0506 (nupC) | A0A0H2XKF0_STAA3 | Pyrimidine nucleoside transport protein | 0 | 3 |
| Oxidoreductase activity | SAUSA300_2146 | A0A0H2XEE2_STAA3 | Zinc-type alcohol dehydrogenase-like protein | 10 | 4 |
|  | SAUSA300_0987 | A0A0H2XJA4_STAA3 | Cytochrome D ubiquinol oxidase, subunit II | 0 | 3 |
|  | SAUSA300_2275 | A0A0H2XK08_STAA3 | Oxidoreductase, short chain dehydrogenase/reductase family | 11 | 4 |
|  | SAUSA300_0179 | A0A0H2XFP0_STAA3 | Formate dehydrogenase (FDH) | 3 | 0 |
| Peroxidase activity | SAUSA300_2418 | A0A0H2XGT6_STAA3 | Alkyl hydroperoxide reductase AhpD | 5 | 0 |
| Phosphopantothenoylcysteine decarboxylase activity | SAUSA300_1764 (epiD) | A0A0H2XJE3_STAA3 | Lantibiotic epidermin biosynthesis protein EpiD | 20 | 0 |
| Prenyltransferase activity | SAUSA300_1153 (uppS) | A0A0H2XGF2_STAA3 | Isoprenyl transferase | 0 | 5 |
| Protein-N(PI)-phosphohistidine-sugar phosphotransferase activity | SAUSA300_2107 (mtlA) | A0A0H2XGP6_STAA3 | PTS system, mannitol specific IIA component | 13 | 2 |
| rRNA (guanine-N7-)-methyltransferase activity | SAUSA300_2644 (rsmG) | RSMG_STAA3 | Ribosomal RNA small subunit methyltransferase G | 0 | 6 |
| rRNA (pseudouridine-N3-)-methyltransferase activity | SAUSA300_0026 (rlmH) | RLMH_STAA3 | Ribosomal RNA large subunit methyltransferase H (RlmH) | 0 | 3 |
| Transaminase activity | SAUSA300_1662 | A0A0H2XGZ7_STAA3 | Aminotransferase, class V | 2 | 5 |
|  |  |  |  |  |  |
| **Other uncharacterized Proteins** | | | | | |
|  | SAUSA300_0303 | A0A0H2XGB8_STAA3 | Putative lipoprotein | 6 | 0 |
|  | SAUSA300_0372 | A0A0H2XH82_STAA3 | Putative lipoprotein | 37 | 8 |
|  | SAUSA300_0992 | A0A0H2XIX8_STAA3 | Putative lipoprotein | 7 | 0 |
|  | SAUSA300_2354 | A0A0H2XFS3_STAA3 | Putative lipoprotein | 0 | 3 |
|  | SAUSA300_0816 | Y816_STAA3 | UPF0337 protei | 19 | 0 |
|  | SAUSA300_1012 | Y1012_STAA3 | UPF0358 protein | 3 | 0 |
|  | SAUSA300_1582 | Y1582_STAA3 | UPF0337 protein | 11 | 3 |
|  | SAUSA300_2622 | Y2622_STAA3 | UPF0176 protein | 0 | 3 |
|  | SAUSA300_0027 | A0A0H2XHW3_STAA3 | Uncharacterized protein | 4 | 0 |
|  | SAUSA300_0031 | A0A0H2XG12_STAA3 | Uncharacterized protein | 3 | 0 |
|  | SAUSA300_0086 | A0A0H2XG96_STAA3 | Uncharacterized protein | 3 | 0 |
|  | SAUSA300_0198 | A0A0H2XFU2_STAA3 | Uncharacterized protein | 4 | 0 |
|  | SAUSA300_0294 | A0A0H2XDV0_STAA3 | Uncharacterized protein | 5 | 0 |
|  | SAUSA300_0298 | A0A0H2XIK1_STAA3 | Uncharacterized protein | 5 | 0 |
|  | SAUSA300_0302 | A0A0H2XKD6_STAA3 | Uncharacterized protein | 4 | 0 |
|  | SAUSA300_0421 | A0A0H2XHM9_STAA3 | Uncharacterized protein | 4 | 0 |
|  | SAUSA300_0518 | A0A0H2XF92_STAA3 | Uncharacterized protein | 3 | 0 |
|  | SAUSA300_0668 | A0A0H2XG53_STAA3 | Uncharacterized protein | 5 | 2 |
|  | SAUSA300_0725 | A0A0H2XHU8_STAA3 | Uncharacterized protein | 6 | 2 |
|  | SAUSA300_0982 | A0A0H2XEX3_STAA3 | Uncharacterized protein | 11 | 0 |
|  | SAUSA300_1230 | A0A0H2XIS0_STAA3 | Uncharacterized protein | 8 | 0 |
|  | SAUSA300_1272 | A0A0H2XEL6_STAA3 | Uncharacterized protein | 0 | 3 |
|  | SAUSA300_1354 | A0A0H2XHL1_STAA3 | Uncharacterized protein | 6 | 2 |
|  | SAUSA300_1506 | A0A0H2XHN9_STAA3 | Uncharacterized protein | 0 | 4 |
|  | SAUSA300_1581 | A0A0H2XG02_STAA3 | Uncharacterized protein | 3 | 0 |
|  | SAUSA300_1698 | A0A0H2XFG6_STAA3 | Uncharacterized protein | 35 | 10 |
|  | SAUSA300_1721 | A0A0H2XIC8_STAA3 | Uncharacterized protein | 0 | 3 |
|  | SAUSA300_1898 | A0A0H2XFB5_STAA3 | Uncharacterized protein | 3 | 0 |
|  | SAUSA300_2077 | A0A0H2XJ44_STAA3 | Uncharacterized protein | 5 | 0 |
|  | SAUSA300_2080 | A0A0H2XIK5_STAA3 | Uncharacterized protein | 0 | 3 |
|  | SAUSA300_2097 | A0A0H2XJE5_STAA3 | Uncharacterized protein | 34 | 7 |
|  | SAUSA300_2381 | A0A0H2XGN1_STAA3 | Uncharacterized protein | 0 | 4 |
|  | SAUSA300_2525 | A0A0H2XFW5_STAA3 | Uncharacterized protein | 6 | 2 |
|  | SAUSA300_2592 | A0A0H2XJ20_STAA3 | Uncharacterized protein | 8 | 0 |
|  | SAUSA300_RS02125 | Q2G1S9_STAA8 | Uncharacterized protein | 0 | 7 |
|  | SAUSA300_RS13415 | A0A0E1VN07_STAA3 | Uncharacterized protein | 3 | 0 |
|  | SAUSA300_0590 | A0A0H2XDU3_STAA3 | Uncharacterized protein | 4 | 0 |
|  | SAUSA300_1204 | A0A0H2XH89_STAA3 | Uncharacterized protein | 3 | 0 |
|  | SAUSA300_1856 | A0A0H2XHR8_STAA3 | Uncharacterized protein | 21 | 6 |
|  | SAUSA300_1858 | A0A0H2XH15_STAA3 | Uncharacterized protein | 3 | 10 |
|  | SAUSA300_1361 | A0A0H2XI67_STAA3 | Uncharacterized protein | 3 | 8 |
|  | SAUSA300_0415 (lpl3) | Y415_STAA3 | Uncharacterized lipoprotein | 6 | 0 |

Comparative GO analysis of the *msaABCR* whole cell proteomics grown under biofilm growth conditions using online tools comparative GO [88, 89]. All proteins differentially expressed ≥ 2 fold is shown.
